# Hydrophobic Interaction Chromatography Coupled to High-Throughput Venomics for Snake Venom Characterisation with Parallel Bioactivity Assessment

**DOI:** 10.3390/toxins18100425

**Published:** 2026-10-01

**Authors:** Tanno Van Herpen, Radja Millenio Kosasih, Jeroen Kool, Julien Slagboom

**Affiliations:** Amsterdam Institute of Molecular and Life Sciences, Division of BioAnalytical Chemistry, Department of 7 Chemistry and Pharmaceutical Sciences, Faculty of Science, Vrije Universiteit Amsterdam, De Boelelaan 1085, 1081 HV Amsterdam, The Netherlands

**Keywords:** high-throughput venomics, Hydrophobic Interaction Chromatography (HIC), coagulation, bioactivity, venom toxins, non-denaturing

## Abstract

Between 80,000 and 140,000 fatalities occur yearly because of snakebite envenoming, which causes it to be classified as a Neglected Tropical Disease (NTD), stressing the importance of snake venom toxin characterisation to aid the development of next-generation antivenoms. Snake venom is made up of a mixture of bioactive proteins that are also known as venom toxins. These venom toxins, being complex and structurally diverse, present a significant challenge for analytical separation and functional characterisation. Traditional methods, such as reversed-phase liquid chromatography (RPLC), often involve denaturing conditions that can alter the native state of these proteins, potentially reducing their bioactivity and limiting subsequent bioassays. Therefore, this study developed and optimised an analytical methodology using non-denaturing Hydrophobic Interaction Chromatography (HIC) for venom separation in conjunction with post-column nanofractionation to perform post-column toxin identification using high-throughput venomics with parallel coagulation bioassays. The results demonstrate that the combination of HIC and nanofractionation allows separation and qualitative profiling of venom toxins with detectable post-column activity, although several toxins co-elute. The post-column bioassays demonstrated detectable coagulation-modulating activity, and high-throughput venomics provided rapid toxin identification capabilities and qualitative information on candidate venom proteins. This approach offers a robust platform for the detailed study of venom toxins, facilitating the discovery of novel bioactive compounds and enhancing our understanding of venom biology.

## 1. Introduction

Venom is used by over 220,000 species, or approximately 15% of the global animal biodiversity, as a means of survival by immobilisation and digestion of prey and as a defence against predators [1]. Each species has adapted its venom to serve specific purposes depending on their surroundings. Snake venoms have primarily been optimised to immobilise prey, but they can also have a pathological effect on other organisms that are not necessarily categorised as prey [1,2]. An estimated 1.8–2.7 million snakebite envenomings have been recorded to occur annually, which, in combination with 80,000–140,000 fatalities and more than 400,000 severe injuries, have caused the World Health Organisation (WHO) to list snakebite envenoming as a Category A Neglected Tropical Disease (NTD) [3,4]. Snakebite envenoming is a significant cause of mortality and morbidity in tropical environments, such as Southeast Asia and sub-Saharan Africa, where it affects the rural and poorer parts of the society much more than wealthier parts of the world [3,5,6].

Snake venoms can exhibit different pathologies, such as neurotoxicity, haemotoxicity and cytotoxicity, which can lead to kidney failure, tissue damage, paralysis and/or haemorrhage. The venomous snakes that are responsible for the majority of fatalities and morbidities are members of the Elapidae (elapids) and the Viperidae (vipers) families. The last of the three families of venomous snakes is the Atractaspidae family. Venoms are complex mixtures of active (poly)proteins or neurotoxins that can disable muscle contraction, cause neuromuscular paralysis and/or have (anti)coagulation effects [3,4].

In this research the focus is on the Viper venoms, which contain proteins that can disrupt tissue integrity, the haemostatic system, and the coagulation system, leading to effects such as necrosis, haemorrhage, and pain. Venom-induced consumption coagulopathy (VICC), a condition often associated with Viperidae species, renders the victim vulnerable to severe and lethal internal bleeding, resulting from the simultaneous action of several venom proteins such as snake venom serine proteinases (SVSPs), phospholipase A2s (PLA_2_s) and snake venom metalloproteinases (SVMPs) [7,8,9,10]. Additionally, viper venoms consist of enzymatic L-amino acid oxidases (LAAOs) and non-enzymatic substances like 3FTxs, Kunitz-type peptides (KUNs), C-type Lectins (CTL or Snaclec) and disintegrins (DISs) [8,10,11,12].

The toxins that are present in viper snake venoms can have potent pathological influences on the haemostasis of the affected organism. SVSPs are primarily haemotoxic and interfere with the complement system the nervous system, blood pressure, blood fibrinogen levels, blood coagulation and platelet aggregation. Many of the activities of SVSPs mimic the functions of the enzyme (pro)thrombin, which plays a significant role in the coagulation cascade [3,13,14]. SVMPs are known to induce haemorrhage and coagulopathy. The SVMPs that affect haemostasis do this by acting as prothrombin activators, exerting effects on platelet aggregation, fibrinogenolysis and blood coagulation. The inclusion of a disintegrin and cysteine-rich domain allows some SVMPs to inhibit platelet aggregation and interfere with the immune response, respectively [3,13,14,15,16]. Viper snake venoms often also contain PLA_2_ enzymes that can induce a wide variety of pathological activities, such as neurotoxic, haemorrhagic, cardiotoxic, myotoxic, and anticoagulant and antiplatelet effects [13,14].

In order to identify bioactive toxins in snake venom analysis research, it is essential that the toxins remain in their native form during the analytical workflow including the chromatographic (or electrophoretic) separation step. As such, they cannot denature as their native form is necessary for the toxins to retain their biological activities so that these activities can be analysed by bioassays (that, e.g., assess a certain pathology). An analytical separation technique that can be used in the non-denaturing separation of toxins in snake venom is Hydrophobic Interaction Chromatography (HIC). Similar to reversed-phase liquid chromatography (RPLC), HIC uses the hydrophobic interactions between the analytes and the stationary phase for separation of the analytes. Where RPLC usually uses acetonitrile or methanol as mobile phases with strong elution strength, which can cause denaturation of susceptible proteins, the mobile phase in a HIC method contains a non-denaturing salting-out salt buffer for gradient elution of proteins [17,18,19,20]. These so-called kosmotropic salts reduce protein solubility, while stabilising the protein structure and making the reversible interactions between the protein’s hydrophobic surfaces and the stationary phase’s hydrophobic groups possible. As a result, the proteins bind to the stationary phase at a high kosmotropic salt concentration and elute at a low salt concentration. Therefore, a gradient from high to low salt concentration is often used for HIC separations [18,19,20]. In several studies where HIC was used for the analysis of intact monoclonal antibodies, the mobile phases contained a phosphate buffer and used ammonium sulphate as the kosmotropic salt [21]. Nevertheless, the use of HIC alone does not demonstrate that every separated protein retains its native structure or full activity.

**Figure 1:**
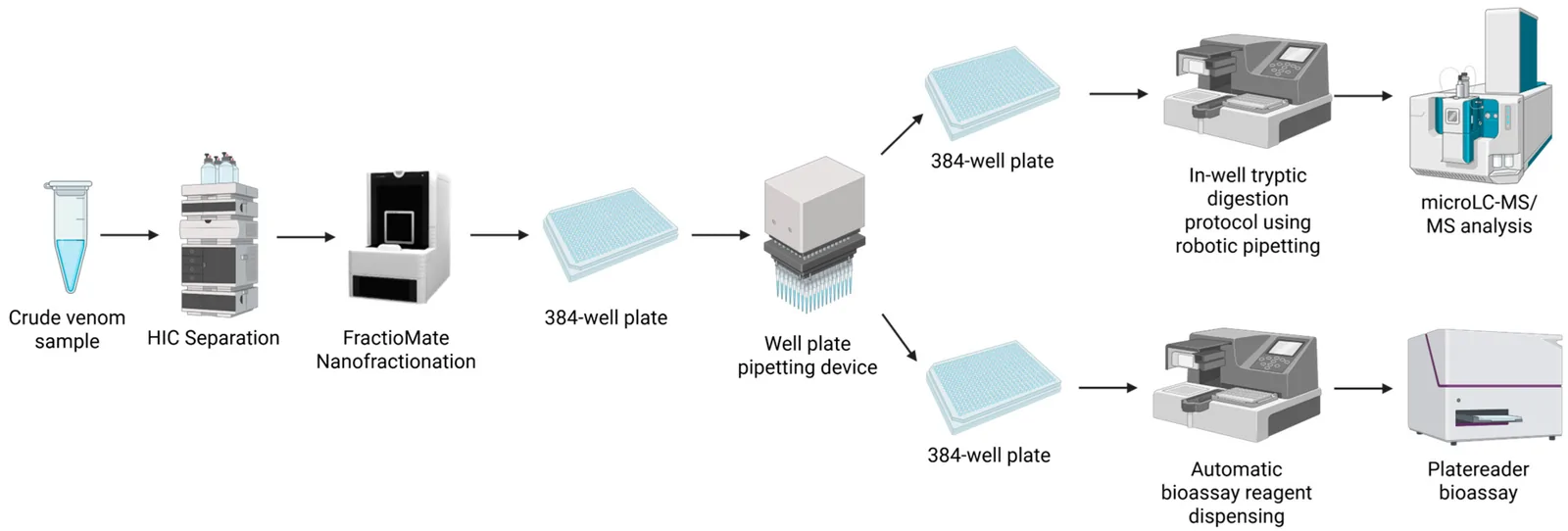
Overview of the nanofractionation analytics workflow central to this study. Crude snake venom is subjected to a HIC separation using high-performance liquid chromatography (HPLC). After separation, the effluent is fractionated into a 384-well plate, which is then duplicated using a well plate pipetting device. One of the duplicated well plates is then used to perform the bioassay to scan for bioactive toxins present in the snake venom. The other well plate is used to perform tryptic digestions followed by microflow liquid chromatography–tandem mass spectrometry (microLC-MS/MS) analysis for proteomics toxin identification by high-throughput venomics.

Post-column profiling of bioactive toxins in snake venom samples can for example be done using a coagulation bioassay previously set up by Still et al. [22,23,24,25,26]. This study focused on RPLC as a separation step thereby likely denaturing (some) bioactive toxins. Instead, HIC could also be used for separation following nanofractionation of the eluting toxins onto a 384-well plate. The fractionated toxins on the well plate can then be duplicated in order to perform both a post-column bioassay for assessing biological activity and high-throughput venomics (HT venomics) for toxin identification. This analytical workflow will be demonstrated in the current study. The bioassay that will be used is a colorimetric coagulation assay which measures a change in the transmittance of the assay mixture in each well over time. Plasma coagulation causes a reduced light transmittance leading to an increased absorbance [26,27]. For HT venomics, 384-well plates with fractionated toxins are subjected to tryptic digestion following measurement of the tryptic digest in each well by microLC-MS/MS. From these analyses, Mascot search results are performed for toxin identifications [5]. The data acquired from the wells can then be processed into so-called Protein Score Chromatograms (PSCs). For this, using scripts, the protein score from Mascot for each toxin found in each well is plotted on the *y*-axis, while the fractionation retention time for each toxin is plotted on the *x*-axis. These PSCs can be overlaid with the LC-UV and chromatographic bioassay data (plotted by plotting fractionation retention time on the *x*-axis versus bioassay response of each fraction on the *y*-axis) in order to tentatively associate candidate toxins with bioactivity at a certain retention time [5]. The nanofractionation analytics workflow is schematically represented in Figure 1. Key focus in the study will be the evaluation and demonstration of the compatibility of the HIC separation, which uses high non-volatile salt concentrations, with post-column bioassaying and HT venomics analysis.

The objective of this study was to develop and apply a non-denaturing analytical separation procedure using HIC with post-column HT venomics and bioassaying for investigation of snake venoms. In this regard, the current study focused on *Bothrops venoms*. The post-column analyses were performed after nanofractionating and collecting the mobile phase after the HIC separation in 384-well plates. As for the proof-of-concept (POC) bioassay, a coagulation bioassay was applied in combination with *Bothrops* viper venoms known to have a significant impact on the coagulation cascade. These venoms contain both procoagulant and anticoagulant components [13,28], and the susceptibility of some procoagulant activities to conventional RPLC provides a rationale for evaluating HIC [27]. The methodological contribution is the integration and evaluation of HIC within the established workflow, rather than the introduction of nanofractionation, automated digestion or PSCs.

## 2. Results

### 2.1. Optimisation of the HIC Gradient and Post-Column Compatibility Assessment of the Coagulation Assay and of HT Venomics

The Hydrophobic Interaction Chromatography (HIC) method was first optimised using *Bothrops jararaca* venom. A systematic evaluation of different gradient profiles is provided in the supporting information (Supplementary Materials Figures S1–S6), where the final gradient (Supplementary Materials Figure S6) was selected based on optimal separation efficiency. This optimised gradient was subsequently applied to *Bothrops jararaca*, *Bothrops neuwiedi*, *Bothrops asper*, and *Bothrops atrox,* for which comparable and chromatographic profiles were obtained. Therefore, a single HIC gradient was used for all venoms and is described in the Materials and Methods section. Following separation, the HIC effluent was nanofractionated onto 384-well plates to enable parallel post-column coagulation bioassaying and high-throughput venomics (HT venomics) of the same toxin fractions.

Compatibility of the HIC eluent with the coagulation bioassay: Because HIC employs high concentrations of ammonium sulphate, its compatibility with the coagulation bioassay was first evaluated. When 5 µL of undiluted HIC effluent was directly combined with calcium chloride and citrated plasma, no coagulation activity was observed at the beginning of the chromatographic run or during the re-equilibration phase. These time windows correspond to the highest salt concentrations in the HIC gradient. As the ammonium sulphate concentration decreased during the gradient, coagulation activity gradually reappeared. Bioactivity became detectable at approximately 1.4 M of ammonium sulphate and was no longer suppressed below approximately 0.5 M. To eliminate salt-induced bioassay inhibition, all fractionated plates were therefore diluted prior to bioassaying.

**Figure 2:**
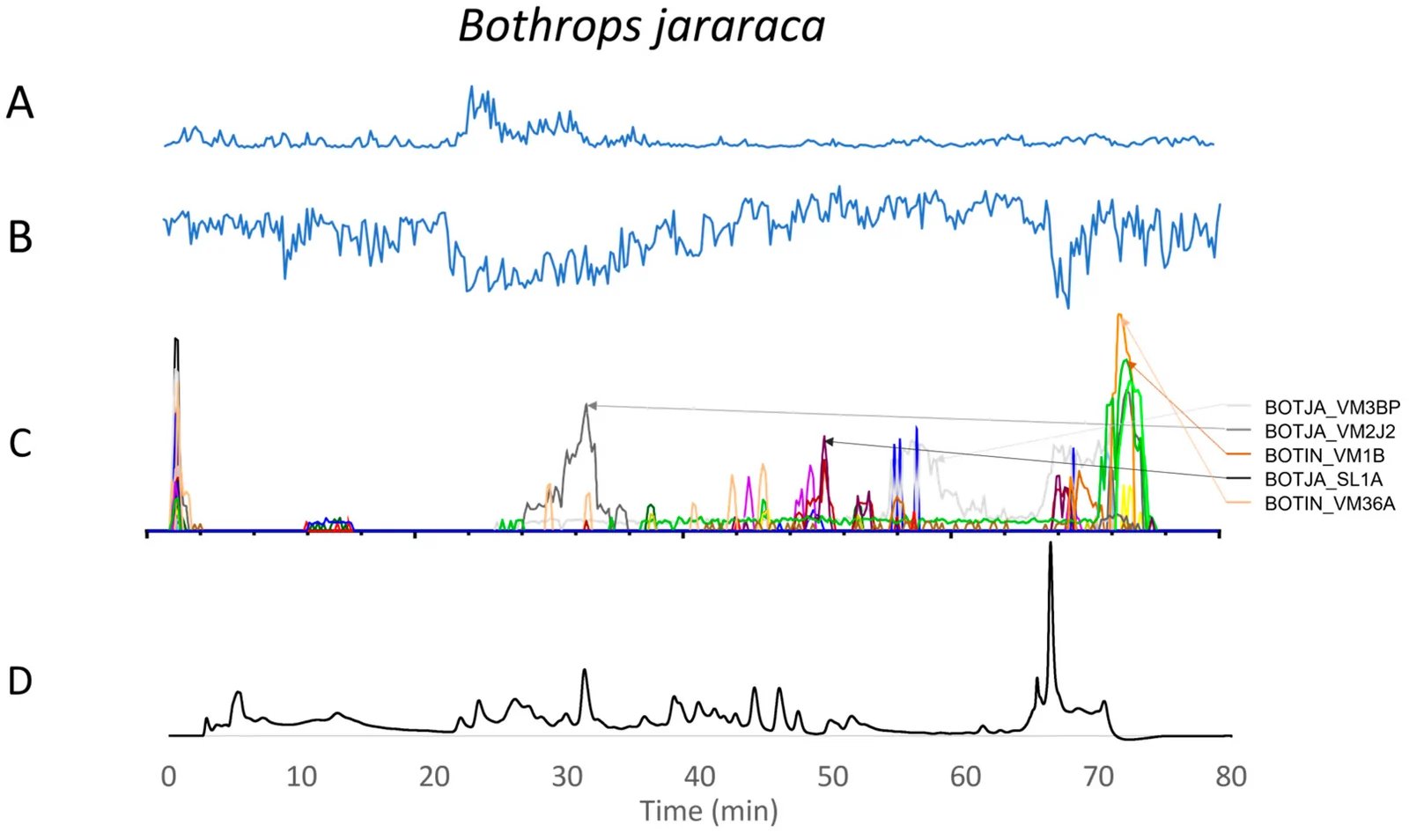
Superimposition of chromatographic bioassay data, HT venomics PSC data, and LC-UV data of *Bothrops jararaca* venom analysis. PSCs and LC-UV data of *Bothrops jararaca* venom toxins identified are below the corresponding bioactivity and LC-UV chromatograms resulting from venom separation using the optimised HIC method. For the results of the bioassay data, a 36-times dilution for cycle 5 (i.e., procoagulation) and a 18-times dilution for cycle 80 (i.e., anticoagulation) are shown. (**A**) Bioactivity chromatogram of cycle 5. (**B**) Bioactivity chromatogram of cycle 80. (**C**) Selected qualitative PSCs. (**D**) LC-UV chromatogram of the HIC separation.

**Figure 3:**
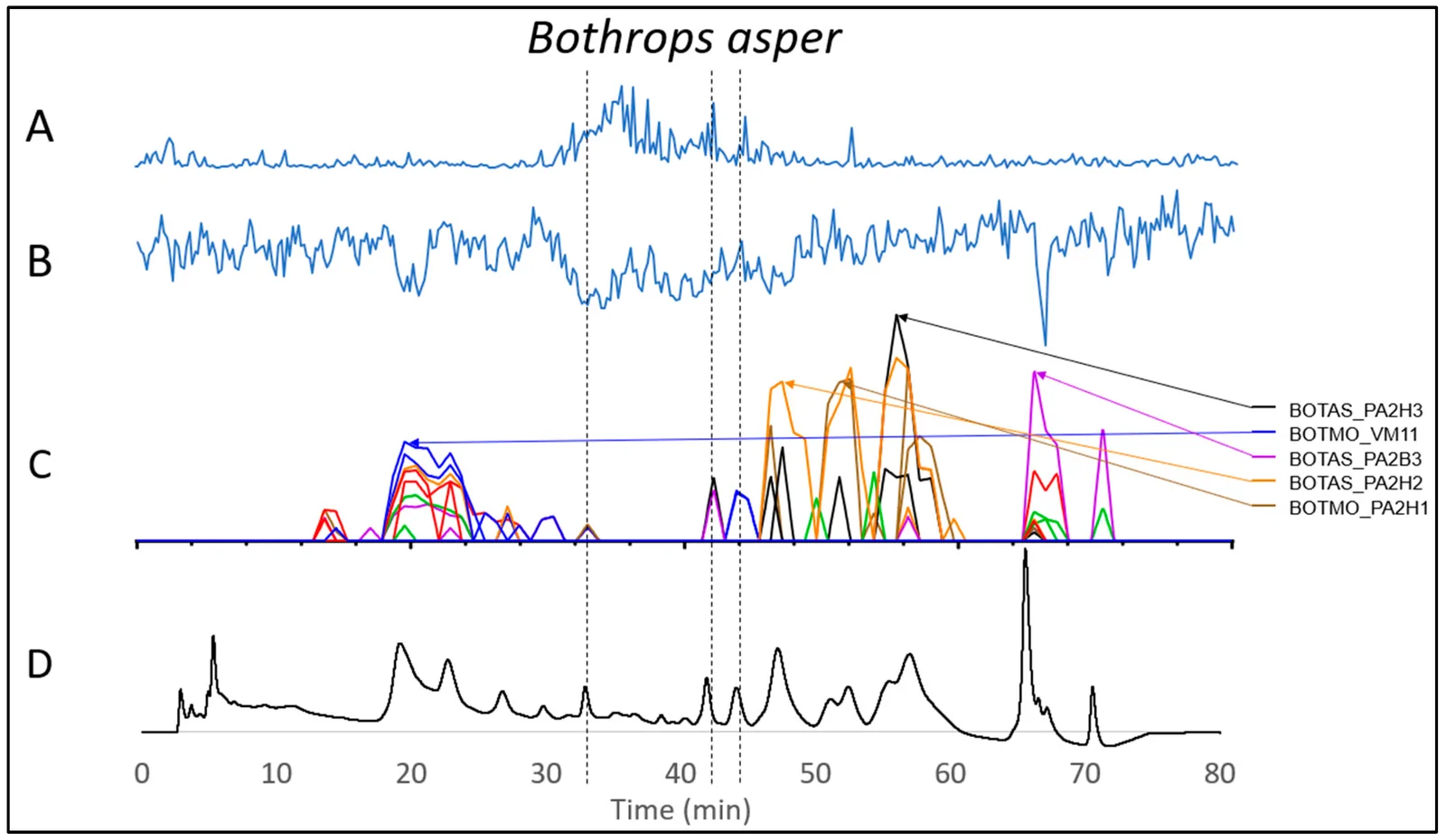
UniProt database Protein Score Chromatograms (PSCs) of the *Bothrops asper* superimposed with the corresponding bioactivity and LC-UV chromatograms after separation using the optimised HIC method. For the results of the bioassays the 144-times dilution for cycle 5 (i.e., procoagulation) and the 18-times dilution for cycle 80 (i.e., anticoagulation) were processed. (**A**) Bioactivity chromatograms of cycle 5. (**B**) Bioactivity chromatograms of cycle 80. (**C**) The Protein Score Chromatograms. (**D**) LC-UV chromatograms of the corresponding HIC-HPLC separations.

**Figure 4:**
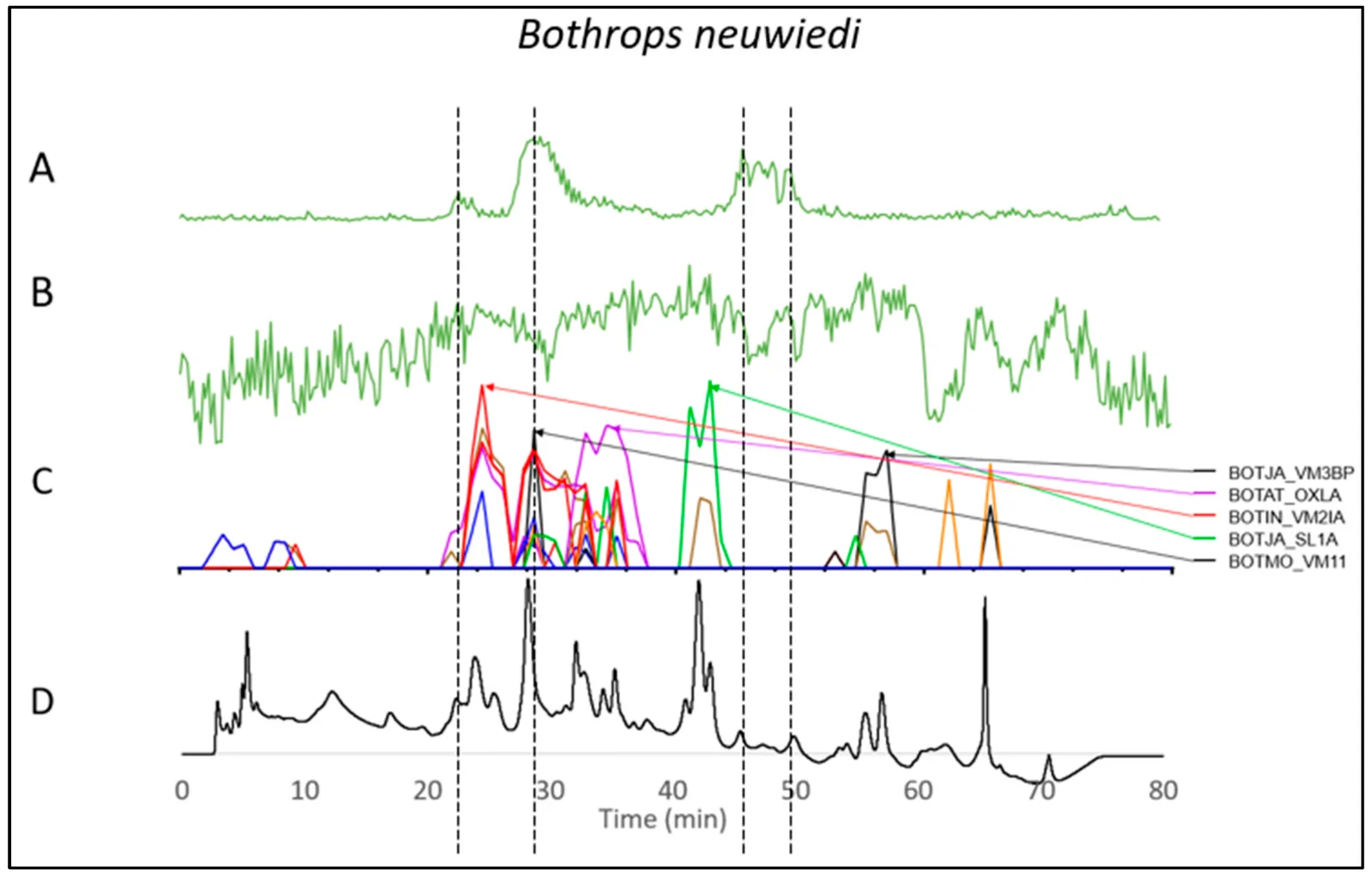
Superimposition of bioassay, venomics and UV data of *Bothrops neuwiedi*. Protein Score Chromatograms (PSCs) of the *Bothrops neuwiedi* superimposed with its corresponding bioactivity and LC-UV chromatograms after separation using the optimised HIC method. For the results of the bioassays of the *Bothrops neuwiedi* the 18-times dilution for cycle 5 (i.e., procoagulation) and the 9-times dilution for cycle 80 (i.e., anticoagulation) were processed. (**A**) Bioactivity chromatograms of cycle 5. (**B**) Bioactivity chromatograms of cycle 80. (**C**) The Protein Score Chromatograms. (**D**) LC-UV chromatograms of the corresponding HIC-HPLC separations.

**Figure 5:**
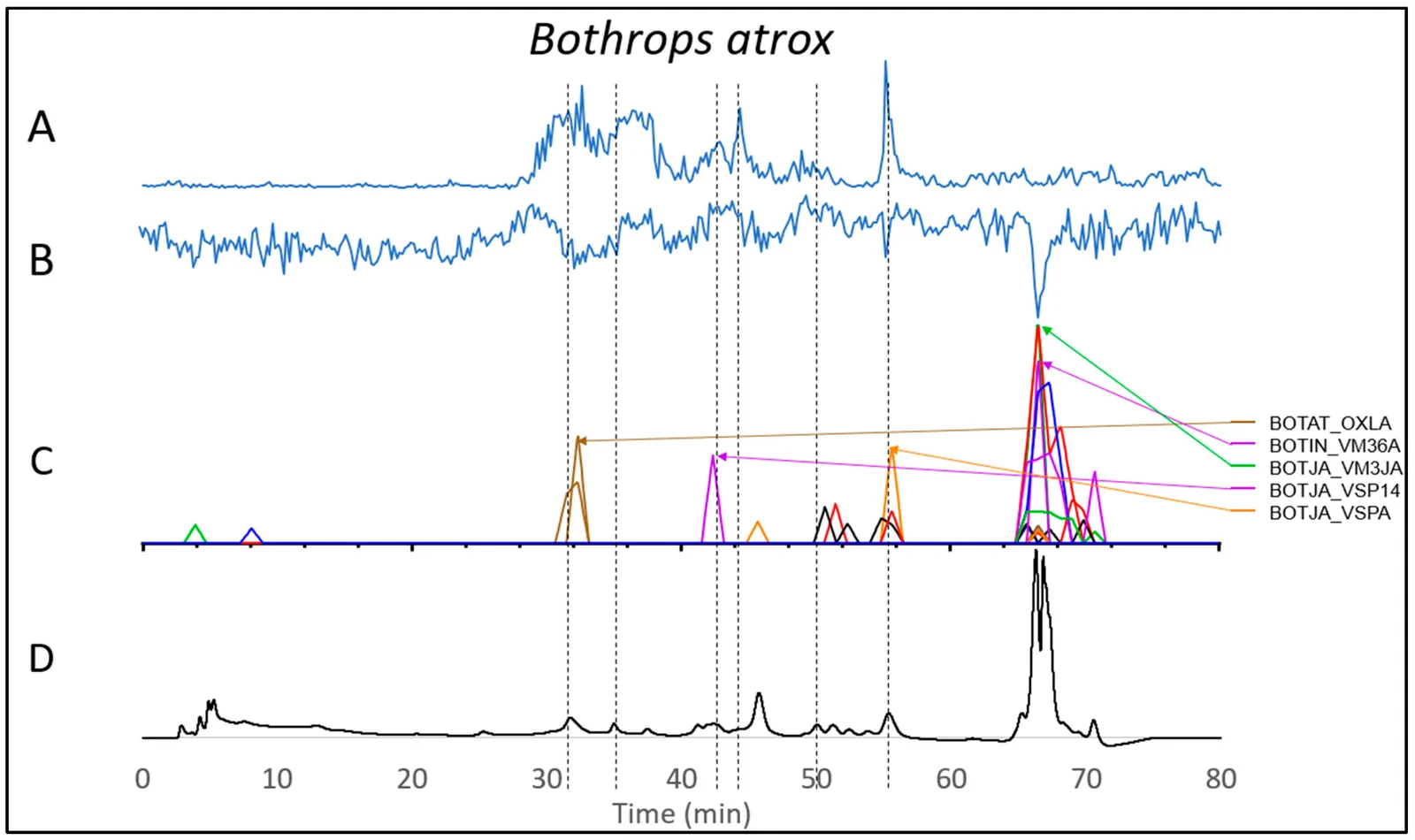
UniProt database Protein Score Chromatograms (PSCs) of the *Bothrops atrox* superimposed with the corresponding bioactivity and LC-UV chromatograms after separation using the optimised HIC method. For the results of the bioassays the 144-times dilutions for cycles 5 and 80 were processed. (**A**) Bioactivity chromatograms of cycle 5 (i.e., procoagulation). (**B**) Bioactivity chromatograms of cycle 80 (i.e., anticoagulation). (**C**) The Protein Score Chromatograms. (**D**) LC-UV chromatograms of the corresponding HIC-HPLC separations.

For the different venoms, distinct optimal dilution factors were obtained (e.g., 18× or 36×). The specific dilution used for each experiment is reported in the corresponding figure captions. Lower dilutions (e.g., 18×) enabled detection of weakly active toxins but often produced broad and saturated peaks for highly active components. Higher dilutions (e.g., 36×) reduced these dominant signals, yielding sharper and better-defined bioactivity peaks and allowing more accurate correlation with LC-UV and HT venomics Protein Score Chromatograms (PSCs). The dilution strategies applied for each venom are indicated in Figure 2, Figure 3, Figure 4 and Figure 5. Across all venoms, anticoagulant toxins predominantly eluted during the low-salt wash step, whereas procoagulant toxins eluted at various positions throughout the analytical gradient. This behaviour was consistently observed for *B. jararaca*, *B. neuwiedi*, *B. asper*, and *B. atrox*.

Compatibility of the HIC eluent with HT venomics: The compatibility of the high-salt HIC fractions with HT venomics was subsequently evaluated. Undiluted fractions (10 µL) were directly subjected to in-well tryptic digestion followed by microLC–MS/MS analysis. No deterioration in MS performance was observed, even after hundreds of sequential injections.

*Protein elution behaviour and database-dependent toxin identification under HIC conditions:* Distinct elution patterns were observed for the major toxin families across all four venoms, with PLA_2_s eluting mainly during the wash step, SVMPs and Snaclecs throughout the gradient, and LAAOs and SVSPs eluting late. These trends are illustrated in Figure 2, Figure 3, Figure 4 and Figure 5 for the most relevant and tentatively assigned bioactive toxins using UniProt-based PSCs. Figure 2, Figure 3, Figure 4 and Figure 5 are discussed in more detail in the next section. PSC plots of all toxins found for each venom using both the UniProt- and transcriptomics-based PSCs can be found in the supporting information (Supplementary Materials Figures S7–S14).

### 2.2. Superimposition of LC-UV Data, Chromatographic Bioassay Data, and HT Venomics Data

After superimposition of the bioactivity and LC-UV chromatograms, the HT venomics PSC data was also superimposed and aligned. The HT venomics data acquired by database searches using the UniProt database for the venom analyses of *Bothrops jararaca*, *Bothrops neuwiedi*, *Bothrops asper*, and *Bothrops atrox* venoms are shown in Figure 2, Figure 3, Figure 4 and Figure 5, respectively. Next to plotting the HT venomics data acquired by database searches using UniProt for producing the PSCs for plotting in the superimposed chromatographic figures, this was also done for the transcriptomics database search results for which the resulting figures can be found in the Supplementary Materials (Supplementary Materials Figures S15–S18) document ‘Supplementary Materials document Hydrophobic Interaction Chromatography coupled to high-throughput venomics for snake venom characterisation with parallel bioactivity assessment’.

After the superimposition of the PSCs (produced from UniProt database searches), candidate toxins associated with coagulopathic fractions could be tentatively identified in the *Bothrops jararaca*, *Bothrops neuwiedi*, *Bothrops asper*, and *Bothrops atrox* venoms for which the superimposed chromatographic results are given in Figure 2, Figure 3, Figure 4 and Figure 5, respectively. Similar figures where the PSCs were produced from the transcriptomics database searches are included in the Supplementary Materials (Supplementary Materials Figures S15–S18). Several important characteristics of the identified protein toxins can be found in the tables in the Supplementary Materials (Supplementary Materials Tables S1–S4). For these tables, chromatographic PSC data from Figure 2, Figure 3, Figure 4 and Figure 5 and from Supplementary Materials Figures S15–S18 has been extracted and tabulated in numerical format. These extracted numerical results include protein ID, protein description, toxin family and subfamily. To further support the data given in this study, additional tables have been provided in the Supplementary Materials to present the measured data in clearly presented tabulated format. Supplementary Materials Table S5 summarises the coagulopathic profiles of *Bothrops jararaca*, *B. neuwiedi*, *B. asper*, and *B. atrox* by listing the main retention timepoints at which pro- or anticoagulant effects were observed in the coagulation bioassay. Supplementary Materials Tables S6–S9 contain the extracted numerical data for each venom, in which bioassay peak retention times are correlated with tentatively identified toxins based on HT venomics PSCs. For each bioactive timepoint, the tables list the toxin family, subfamily and sub-subfamily, protein identifiers, theoretical biochemical or biological effects (derived from UniProt), and the observed bioassay effect. These tables therefore provide the numerical framework for interpreting the superimposed chromatographic figures (Figure 2, Figure 3, Figure 4 and Figure 5 and Figures S15–S18) and enable direct comparison between experimental bioactivity and database-based toxin annotations.

## 3. Discussion

### 3.1. Methodological Contribution and Compatibility

RPLC-based nanofractionation has previously been coupled to coagulation screening and proteomic toxin identification [28]. The HT venomics workflow subsequently established automated digestion and PSC-based comparisons across fractions [5]. Co-elution and incomplete assignment of activity to individual proteins were also limitations of these earlier approaches. The present study extends the workflow to HIC and evaluates downstream measurements in the presence of its salt-rich eluent. It does not include a matched HIC–RPLC experiment, so differences between published profiles cannot establish superior resolution or activity recovery. Such comparisons would require the same venom preparation, loading, fractionation intervals and assay conditions.

Non-denaturing fractionation has also been explored using size-exclusion chromatography for proteolytic venom activity profiling [29]. HIC is therefore an additional separation option rather than the first non-denaturing approach to post-column venom bioassaying. Its value here is the ability to locate coagulation-modulating fractions and prioritise candidate proteins for subsequent characterisation.

Since HIC uses very high concentrations of non-volatile salts in the mobile phase to achieve analytical separation, this can result in compatibility issues with both the coagulation bioassay and the mass spectrometry measurements required for the HT venomics. Compatibility between the eluent salt concentrations and the post-column coagulation bioassay was assessed by straightforward dilution of the fractions collected on the 384-well plates in order to minimise the effects of the salts on the bioassay performance. As coagulopathic toxins in *Bothrops venoms* are known to be highly active, dilution can retain detectable responses, although it may obscure weaker activities. Peptide identifications were obtained from the HIC fractions, but the absence of obvious deterioration in performance does not quantify ion suppression or establish complete salt removal.

### 3.2. Toxin–Activity Associations and Qualitative Limitations

The venoms of *Bothrops jararaca*, *Bothrops neuwiedi*, *Bothrops asper*, and *Bothrops atrox* were analysed using the complete analytical workflow and the comprehensive results obtained will be discussed next. For correlation of anti- and procoagulant bioactivity peaks with the HT venomics data, the database searches performed were done using both the UniProt (only Serpentes) database and transcriptomics databases for *Bothrops asper* and *Bothrops jararaca*. Additionally, for the database searching of the venomics data using the UniProt database, transcriptomics database search results were obtained by searching the microLC-MS/MS data using these specifically composed databases designated *Bothrops asper* and *Bothrops jararaca*. From this point onwards, whenever UniProt is mentioned, the proteomics database of UniProt was used for the database searches, and when transcriptomics is mentioned, an additional transcriptomics database was used for the database searches. The transcriptomics database value comes from the exact mRNA sequences therein corresponding to the venom toxins to be analysed from the species represented in the reference database. The UniProt database has its values from the extensive data it contains on venom toxins of many venoms in general and as such is capable of also picking up venom toxins from similar venomous species which have amino acid sequences that are the same as those of the toxins found in the species under study (i.e., when the tryptic digests contain peptides which are the same as those found in venoms of other similar species). In addition, when transcriptomics databases are not available for a venom under study, one has to rely on available databases such as UniProt. In addition, UniProt searches also retrieve known information on the snake venom toxins found in many cases including a multitude of references to the existing literature, which helps among other aspects with the identification of proteins. A disadvantage of the UniProt searches can imply the generation of hits on toxins that are similar to the analysed *Bothrops* toxins but that are found in snake venoms from other species. The used transcriptomics databases provide complementary sequence matches, but these do not independently validate the species-specific identity of every toxin. Therefore, the complementary information obtained from both databases will be looked at and discussed in this study. The UniProt database results will be shown here in the main paper. The transcriptomics database results can be found in the Supplementary Materials document.

As several bioactivity peaks were rather broad and from the PSC data, during these retention time intervals several toxins were identified to be eluting, in these cases only toxin IDs could tentatively be assigned to a bioactivity observed. The tables can be found in the Supplementary Materials as Table S6–S9. Using these datasets, correlations will be drawn between the observed biochemical effects of the bioassay, the HT venomics PSC and LC-UV data, and toxin biochemical/biological effects extracted from the UniProt database. This will be discussed in the following paragraphs, venom by venom.

In Figure 2, the combined chromatographic results of the analysis of *Bothrops jararaca* venom are shown. Three retention timepoints were identified to contain bioactive toxins and all showed procoagulant effects. At 23 min, neither the UniProt nor the transcriptomics database identified any toxins. At 30 min, the UniProt database retrieved a single SVMP, which was confirmed by the transcriptomics database. This SVMP is known as jararafibrase-2 and has proven platelet aggregation inhibition characteristics as is noted in the UniProt database. However, at 30 min, a C-type Lectin was identified in the transcriptomics database as well. This C-type Lectin, known as a Snaclec, also has the capability to cause the observed procoagulation effects in the bioassay. At 36 min, a similar situation was observed. At this timepoint bothropasin was identified using the UniProt database, which was substantiated by three SVMP results retrieved by the transcriptomics database shown in Supplementary Materials Table S12. The procoagulation observed could be caused by the Snaclec protein Bfon18 identified using the UniProt database, which is also classified as a Snaclec protein with haemostasis-impairing capabilities.

The combined chromatographic results of *Bothrops neuwiedi* venom analysis can be found in Figure 4. At 24 min, five different SVMPs were identified by the UniProt and the transcriptomics databases. Two of these SVMPs called BnP1 and BnP2 have fibrinogenolytic capabilities according to the UniProt database. Two other SVMPs named batroxstatin-1 and leucurolysin-A in Supplementary Materials Table S7 are known to have platelet aggregation-inhibiting capabilities as deduced from the UniProt database again. The last and fifth SVMP found was insularinase-A, which is known to be a blood coagulation cascade activator, and the other found proteins did not have any known coagulation influences. Insularinase-A is therefore a candidate for the observed procoagulation, but this association does not establish causality, which is also presented in Supplementary Materials Table S7. Furthermore, at 28 min, ten proteins were identified using the UniProt database, of which five were SVMPs, two were SVSPs, two were Snaclec proteins, and one was a CRISP-V toxin. Using the transcriptomics database, three SVMPs and two Snaclec proteins were confirmed (see Supplementary Materials Table S13). Because of the number of identified closely co-eluting toxins, a specific determination of the toxins responsible for the bioactivity observed is difficult. Therefore, a specific identification of the bioactive protein(s) is not possible for the bioactive peak at 28 min with the current data. At the 46 and 50 min retention timepoints no proteins were identified using the UniProt and the transcriptomics database.

Looking at the superimposed chromatographic results for the *Bothrops asper* venom analysis (Figure 3), four retention timepoints were identified as procoagulant with an additional single negative peak showing anticoagulant properties. At 20 min, the protein appears to exhibit weak anticoagulant effects. A procoagulant contribution was hypothesised based on the known functions of the identified proteins, but this cannot determine the interpretation of the measured negative signal. This is because five identified toxins are known to have fibrinogenolytic properties, a single protein is known to be a platelet aggregation inhibitor (leucurolysin-A), and another protein has been described as a blood coagulation cascade activator (basparin-A) according to UniProt. The hypothesis about the seemingly weak anticoagulant activity relates to sudden and fast coagulation after the effects of the procoagulation. Bioactive toxins have sorted their effects by the rapid depletion of clotting factors involved in coagulation. In other words, because of the fast procoagulation effects around the 20 min retention time frame, initial clot formation that is very fast could result in microclots that do not continue to form the eventual uniform jelly clots in the wells but instead yield floating microclots in the wells. This in turn could result in lower absorbance being measured in these wells than in those wells where normal coagulation has taken place. This could be observed as a negative signal in the anticoagulation chromatograms, but the current measurements do not establish this mechanism. Therefore, rapid procoagulation is a possible explanation for the negative endpoint response at the retention time frame of 30 to 60 min, rather than a demonstrated mechanism. Timepoints 33, 42, and 44 showed procoagulation for which a Snaclec protein, an L-amino oxidase, and an SVSP, respectively (see Supplementary Materials Table S8), were found from the UniProt PSC data, which was substantiated by the presence of the same protein family in the transcriptomics PSC results (see Table S10).

When looking at the superimposed chromatograms of the *Bothrops atrox* venom data, there are seven retention timepoints of interest, of which the last one at 66 min was the only one with anticoagulation properties. After 32 min, where procoagulant signals were observed, the UniProt database retrieved two L-amino-oxidases that it identifies as two possible blood coagulation cascade activators. The 36-, 42-, and 50-min retention timepoints did not retrieve significant results from the UniProt database, but the 50 min timepoint did give the SVMP batroxstatin-2 from the transcriptomics database results. At 42 min, the SVSP HS114 was found in the UniProt database where the protein is known to have fibrinogenolytic activity. At 56 min, the SVSP bothrombin elutes and is known to have platelet aggregation-activating and blood coagulation cascade-activating effects; another SVSP BthaTL is known as a haemostasis-impairing toxin. Furthermore at 56 min, the SVMP basparin-A has been described by UniProt as having coagulation cascade-activating properties as well, which could indicate a double activation by the co-eluting proteins.

Supplementary Materials Tables S10–S13 are given in the Supplementary Materials document to provide the transcriptomics-based toxin identifications that complement the UniProt-based correlations shown in Supplementary Materials Tables S6–S9. These tables link bioactivity peak retention times to venom gland transcript sequences in the additional databases, providing complementary candidate assignments rather than independent species-specific validation of the HT venomics identifications. For each bioactive retention timepoint, the tables list the transcript accession or code, assigned toxin family, protein abbreviation, and full protein name. In cases where multiple transcripts co-elute, all candidates are reported. Together, Supplementary Materials Tables S10–S13 enable direct comparison between UniProt-based and transcriptomics-based toxin annotations and support comparison of candidate sequences with fraction-level pro- and anticoagulant activities.

Overall, some of the protein toxin candidates associated with coagulopathic effects had matching toxin IDs from the HT venomics data thereby tentatively identifying them. Responsible toxins for some bioactivity chromatogram peaks could however not be determined. In the first published HT venomics study by Slagboom et al., it was shown that in several cases multiple toxin peaks were found for the same toxins observed in the PSCs of these toxins. As this study also acquired direct LC-MS analysis on the separated toxins, accurate toxin masses were also determined. This could be done in a hyphenated LC-MS fashion since reversed phase was used for the chromatographic separations, which is well-suitable for LC-MS analysis of intact masses of eluting toxins. Slagboom et al. found that differences in accurate intact toxin masses that were being measured for the same toxins identified by HT venomics could be attributed to the same toxins with different post-translational modifications (PTMs) eluting at different retention times [5]. Some of these PTMs could be contributed to the occurrence of different glycosylations on the same toxins thereby changing the molecular weight of these toxins, but not their toxin IDs assessed by HT venomics. As glycosylations can have significant influences on the retention behaviour of venom toxins when they are separated by HIC, these glyco-proteoforms of the same toxins are expected to have clear differences in retention time during the HIC analyses (as glycosylations heavily influence toxin stability and solubility) [30]. It is observed that for different toxins found in the PSCs for the venoms analysed, multiple peaks were found in the HIC PSC traces that could be consistent with this, but the present data do not establish the responsible modifications.

PSCs indicate where peptides compatible with database proteins were detected. Mascot scores are not calibrated measures of toxin concentration, relative abundance or potency. Differences in peptide detectability, sequence coverage, shared peptides and database representation can affect score profiles. Accordingly, a large PSC peak does not establish that a toxin predominates in a fraction or causes its biological response. Although PSC-derived measures have been explored for compositional comparisons in earlier HT venomics work [5], this study uses PSCs only for qualitative localization of sequence matches. No quantitative toxin-composition analysis was performed.

Quantitative approaches such as emPAI or NSAF could help estimate fraction composition, but abundance alone would not establish causality because toxins differ in potency and can have opposing or interacting effects. Quantification and validation of its performance fall outside the present proof-of-concept study. The supplementary tables therefore report candidates and their reference annotations separately from the measured response of the fraction. Platelet-modulating annotations, in particular, cannot by themselves explain the response in this plasma coagulation assay. Further purification and functional testing, or appropriately controlled inhibition experiments, are needed to confirm individual toxin–activity assignments.

HIC did not resolve all venom components. Several active regions contained multiple candidates, and some fractions had activity without an assigned protein. Salt dilution reduced matrix interference but also reduced toxin concentration, potentially obscuring low-abundance or weakly active components. Detectable activity after HIC does not demonstrate complete recovery of every toxin or preservation of native structure; neither was it quantified. Orthogonal separation by ion-exchange or size-exclusion chromatography, or further optimisation of HIC selectivity, may improve candidate resolution. Each additional step would require assessment of recovery and downstream assay compatibility.

### 3.3. Scope and Extension to Mixed Venoms

The four venoms represent one genus, and the results cannot establish performance across all venom types. Neurotoxic and haemotoxic activities are not mutually exclusive. The neurotoxic PLA_2_ ammodytoxin A can bind factor Xa and inhibit prothrombinase, providing an example of a toxin with both neurotoxic and anticoagulant properties [31]. A predominantly neurotoxic venom would therefore not automatically be a negative control for coagulation. These activities also cannot be generalised to every PLA_2_.

Application to mixed neurotoxic and haemotoxic venoms would require complementary neurotoxicity assays, together with verification that the relevant proteins are recovered and sufficiently separated. The present plasma assay measures clotting-related effects and does not measure neurotoxicity. Opposing effects from co-eluting components may mask activity, while poorly represented sequences may remain unidentified. Mixed-venom studies and matched comparisons with alternative separations are therefore future validation steps rather than demonstrated capabilities of the current workflow.

## 4. Conclusions

During this study four *Bothrops* snake venoms have been successfully analysed using an approach integrating HIC separations post-column via nanofractionation to both coagulation bioassays and HT venomics. The compatibility issues of high concentrations of non-volatile salts that are used during the HIC separations with both the coagulation bioassay and the MS analyses have been evaluated, with detectable post-column activity and peptide identifications obtained under the conditions used. The analytical methodology demonstrated in this study and the findings from the venoms analysed open new avenues for studying and understanding the function(s) of bioactive toxins in venoms. This is a qualitative proof of concept: co-elution prevents definitive assignment of every fraction-level response to an individual toxin. The results do not establish complete preservation of native structure or activity, or performance across other venom types. Further work should compare separation and activity recovery under matched conditions, improve candidate resolution through complementary separations, and validate selected toxin–activity associations with functional assays. Application to mixed neurotoxic and haemotoxic venoms will require additional assay and recovery validation.

## 5. Materials and Methods

### 5.1. Chemicals

Milli-Q water was obtained using a Milli-Q plus water purification system (Millipore, Amsterdam, The Netherlands). Sodium phosphate dibasic (ACS reagent, ≥99.0%), sodium phosphate monobasic monohydrate (ACS reagent, ≥98%), ammonium bicarbonate (eluent additive for LC-MS), calcium chloride dihydrate (ACS reagent, ≥99.9%), hydrochloric acid (ACS reagent, 37%), 2-mercaptoethanol (≥99.0%), iodoacetamide (BioUltra, ≥99.0%), and trypsin from bovine pancreas (lyophilised powder, ≥95%) were obtained from Sigma-Aldrich (Darmstadt, Germany). Sodium hydroxide (≥99%, pellets for analysis) and ammonium sulphate (≥99.5%) were supplied by Merck KGaA (Darmstadt, Germany). Furthermore, formic acid (ULC-MS-CC/SFC grade, 99%), 2-propanol (HPLC grade), and methanol absolute (HPLC grade) were supplied by Biosolve (Valkenswaard, The Netherlands). Lastly, Bovine Serum Albumin plasma (with Sodium Citrate, sterile-filtered) was purchased from Biowest (Nuaillé, France) and the ESI-L low-concentration tuning mix for mass spectrometry calibration was obtained from Agilent (Santa Clara, CA, USA).

### 5.2. Snake Venoms

In this study, the venoms of four species of the Viperidae family were used. The *Bothrops asper* and *Bothrops atrox* venoms were sourced from the historical VU venom collection. *Bothrops jararaca* and *Bothrops neuwiedi* venoms were provided by Latoxan (Portes-lès-Valence, France). The snake venoms were stored in a lyophilised form at 4 °C, until 5 mg/mL stock solutions were made by reconstitution in Milli-Q (which were stored at −80 °C).

### 5.3. HIC Separation

An Agilent 1260 Infinity II HPLC system (Agilent Technologies, Santa Clara, CA, USA) consisting of a 1260 Multisampler G7167A, a Quaternary Pump G7111B and a Variable Wavelength Detector G7114A was used during the course of the study. The column that was used during the HIC separations was an Agilent AdvanceBio HIC HPLC column (Agilent Technologies, Santa Clara, CA, USA) (100 mm × 4.6 mm) with a particle size of 3.5 μm and a pore size of 450 Å. The HIC separation made use of two mobile phases that followed a gradient for the separation of the snake venoms. Mobile phase A consisted of a 50 mM sodium phosphate buffer and mobile phase B consisted of a 50 mM sodium phosphate buffer with the addition of 2 M of ammonium sulphate. Both mobile phases used were at a pH of 7.0. The used gradient started at 5% mobile phase A, which was increased to 75% over a period of 60 min. Next, the concentration of mobile phase A was increased from 75% to 100% in 1 min, which marked the start of the washing step. After 5 min of washing the column on 100% for mobile phase A the concentration was brought back down to 5% mobile phase A in a time span of 4 min, where it stayed for 10 more minutes in order to re-equilibrate the column. A constant flow of 0.5 mL/min was used throughout the entire method. The detector used to record the LC-UV chromatograms of the analysed snake venoms was operated at the wavelengths of 220 nm and 254 nm. The HIC column was kept at an oven temperature of 30 °C. The used seal wash was composed of 15% isopropanol in Milli-Q and the injection wash consisted of a 50/50 ratio of methanol and Milli-Q. An injection volume of 100 µL was applied using stocks of 0.5 mg/mL solutions of the snake venoms, corresponding to 50 µg venom per injection.

### 5.4. Fractionation and Plate Duplication

After separation and UV detection, the effluent of the chromatographic HIC separations was fractionated in high resolution using a FractioMate™ (Vrije Universiteit, Amsterdam, The Netherlands) controlled by Fractiomator software 2.0.0.16 and the nanofractions were collected on 384-well clear flat-bottom microtitre plates from Greiner Bio-One (Alphen aan den Rijn, The Netherlands). The FractioMate fractionated each entire HIC gradient on a 384-well plate using a 12 s collection time for each well, in serpentine fashion. The 384-well plates with fractionated toxins can be stored at −20 °C or can directly be used to make duplications for HT venomics and the coagulation bioassay. The well plates were then duplicated using a Rainin MicroPro 300 robotic pipet (Mettler-toledo, Tiel, The Netherlands). For the bioassay, 5 µL from each diluted fraction (Section 5.5) was transferred to the new plate and for HT venomics, 10 µL of each of the wells from the stock plate was transferred to a new plate. Subsequently, for the bioassay and for HT venomics, additions of (bio)chemicals were performed using a Multidrop 384 Reagent Dispenser (Thermo Fisher Scientific, Ermelo, The Netherlands) (see the next sections for the bioassay and tryptic digestion protocols).

### 5.5. Coagulation Bioassay

Fractions were diluted ninefold by transferring 10 µL from each nanofraction into 80 µL of Milli-Q water, followed by additional serial dilutions as required. From these diluted plates, 5 µL was transferred to the bioassay plate to determine the minimal dilution that restored full assay performance. The factors 9×, 18×, 36× and 144× refer to dilutions relative to the collected fraction before addition of the assay reagents.

A detailed description of the coagulation bioassay is provided by Still et al. [26]. In brief, once the duplicated well plates were made the bioassay was performed on one of the well plates. A Multidrop 384 Reagent Dispenser was used to first pipet 50 µL of a 20 mM calcium chloride solution onto each of the well plates, followed by the addition of 50 µL of citrated bovine plasma. Thereafter, a CLARIOstar Plus (BMG Labtech, Ortenberg, Germany) microplate reader was used to measure the absorbance of each well at a wavelength of 595 nm. Furthermore, the plate reader used an orbital scan to measure the absorbance using 8 flashes per cycle, 80 cycles in total and 119 s per cycle. The measured absorbance in each well was then plotted (on the *y*-axis) against the retention time of fractionation (on the *x*-axis) to obtain the bioactivity chromatograms. For the bioactivity chromatograms, two timepoints of the kinetic measurement were taken. For plotting the procoagulation bioassay chromatograms, kinetic cycle measurement 5 was used. For plotting the anticoagulation bioassay chromatograms, kinetic cycle 80 (i.e., the endpoint measurement) was used. During cycle 5 the coagulation has just started, which makes it possible to determine fractions with procoagulant activity (observed as a positive signal compared to the control). During cycle 80 the coagulation is almost complete and therefore fractions with anticoagulant activity can be detected (observed as negative signal compared to the control).

### 5.6. In-Well Tryptic Digestion

Tryptic digestion procedures were performed on duplicated 384-well plates for proteomics analysis. Using the Thermo Fisher Multidrop 384 Reagent Dispenser, 25 µL of the reduction buffer, which consisted of 0.05% beta-mercaptoethanol in digestion buffer, was added to each of the wells. The digestion buffer was made up of a 50 mM ammonium bicarbonate solution at a pH of 8.2. This plate was then incubated at 80 °C in a Hewlett Packard HP 6890 GC System (Hewlett-Packard, Wilmington, DE, USA) for 40 min and afterwards the plates were allowed to cool down. Subsequently, 10 µL of alkylation reagent solution (75 mM iodoacetamide in Milli-Q) was added to the wells and the plates were incubated in the dark, at room temperature for 30 min. The next step was to add 10 µL of a 0.01 µg/µL trypsin in digestion buffer solution to each well after which the plates were incubated overnight at 37 °C. The final part of the digestion was to quench the reactions using 10 µL of a 1.25% formic acid solution in Milli-Q for each well.

### 5.7. MicroLC-MS/MS Analysis

For the microLC separations of the tryptic-digested venom toxin samples on the well plates, a Thermo Fisher UltiMate 3000 RSLCnano system (Rockford, IL, USA) was used. The autosampler that was used had the capacity to hold up to four well plates and could inject directly from the 384-well plate into the analytical system using a partial-loop injection mode. The contents of the wells were injected into the microLC system in the same serpentine fashion sequence as that was used during the fractionations of the HIC separations. The separations were performed using a Kinetex 2.6u XB-C18 LC Column (Phenomenex, Torrance, CA, USA) (150 mm × 0.3 mm) with a particle size of 2.6 μm and a pore size of 100 Å. The separation made use of two mobile phases with mobile phase A consisting of 98% Milli-Q, 2% acetonitrile and 0.1% formic acid, and mobile phase B consisting of 98% acetonitrile, 2% Milli-Q and 0.1% formic acid at a flow rate of 6 µL/min. The method made use of a gradient that started at 5% mobile phase B, which increased to 40% mobile phase B in 4 min. In 0.2 min, the concentration for mobile phase B was then increased to 90%, where it stayed for 0.8 min after which it decreased to 5% mobile phase B for column equilibration which lasted 1.5 min. The column was kept at a constant 30 °C in the column oven.

After microLC separation, mass detection was performed by a Sciex 7600 ZenoTOF mass spectrometer (SCIEX, Framingham, MA, USA) with a Sciex OptiFlow 1–50 μL Micro/MicroCal ion source. During the mass detection the ionisation start time was set at 2.5 min to exclude the initial part of the microLC run from acquisition. After 2.5 min the spectral data was gathered in the range of 100–2000 Da with an accumulation time of 0.01 s using CID (Collision-Induced Dissociation) in Information-Dependent Acquisition (IDA) mode. A mass tolerance of 50 mDa was set in the IDA mode next to a maximum of 45 candidate ions, an intensity threshold that exceeds of 300 counts/s, with no dynamic background subtraction with exclusion of former candidate ions for 6 s and after one occurrence. The declustering potential was set to 80 V with a spread of 0 V and a collision energy spread of 0 V as well. The dynamic CE was used during the analysis with a maximum of 80 V and a minimum of 5 V, where the charge states 2 to 5 were selected and the selected isotope was monoisotopic. The ESI source was used in the positive ionisation mode; the source temperature was set to 150 °C, and the capillary voltage was set to 1.2 kV. Furthermore, the curtain gas was set to 35. CAD gas was set to 7; the ion source gas 1 was used at 15 psi and the second ion source gas was used at 20 psi. For more details the study of Slagboom et al. can be consulted [32]. The MS data was obtained was processed into MGF files using ProteoWizard’s msConvert version 3.0.24050. The resulting MGF files were used for database searches with Mascot (Matrix Science, London, UK) via Mascot Daemon, enabling batch processing of all files simultaneously. Searches were performed in February 2024 against the UniProtKB/Swiss-Prot database restricted to entries from the taxonomic group Serpentes, using Snakes2023_20230329.fasta containing 2712 reviewed entries [33], as well as against additional venom gland transcriptomic datasets. The additional databases used for these searches were designated *Bothrops asper* and *Bothrops jararaca*; a published *B. jararaca* transcriptomic resource is described by Junqueira-de-Azevedo et al. [34]. The search date is not a verified download date or database release. For the searches, the following search parameters were used: for the instrument type an ESI-QUAD-TOF was selected, as digestion enzyme Trypsin with the allowance of one missed cleavage, as fixed modification carbamidomethyl on cysteine and as variable modifications amidation (protein C-terminus) and oxidation of methionine. The fragment mass tolerances were set to an MS/MS tolerance of ±0.05 and a peptide tolerance of ±0.2 Da.

Using different scripts that were produced in R, the results were extracted from the Mascot search logs and filtered for all relevant information (including species, toxin ID, peptides identified, protein score, sequence coverage, etc). Protein scores from each detected venom protein from each well were plotted on the *y*-axis against the corresponding retention times of fractionation on the *x*-axis, and a combination of all resulting plots was then produced into a single Excel file. These steps generated the so-called Protein Score Chromatograms (PSCs), which show the proteins (i.e., toxins) that have been detected in a format that can be superimposed with the LC-UV chromatograms and the chromatographic bioassay data. The R scripts and a more detailed description of the data processing methods can be found in the study of Slagboom et al. [5].

## Data Availability

Raw proteomics data are publicly available in Zenodo at https://doi.org/10.5281/zenodo.21224087. The figures and tables supporting the qualitative interpretation are provided in the manuscript and Supplementary Materials.

## Supplementary Materials

The following supporting information can be downloaded at https://www.mdpi.com/article/10.3390/toxins18100425/s1, Figures S1–S6: HIC gradient development; Figures S7–S14: Protein Score Chromatograms from UniProt and additional database searches; Figures S15–S18: Additional database-search PSCs overlaid with LC-UV and coagulation profiles; Tables S1–S4: Protein nomenclature and annotations; Table S5: Reported coagulation-response regions; Tables S6–S13: Candidate proteins associated with fraction retention times. The supplementary document accompanies this manuscript.
