# Supplementary material for: Hydrophobic Interaction Chromatography Coupled to High-Throughput Venomics for Snake Venom Characterisation with Parallel Bioactivity Assessment
Source: Toxins (Basel). 2026 Oct 1;18(10):425. doi: 10.3390/toxins18100425 (PMC13709914; doi:10.3390/toxins18100425)
Supplement: Supplementary file 1 [file toxins-18-00425-s001.zip › toxins-4452344-supplementary.pdf]

Supplementary Materials: Hydrophobic Interaction Chromatography Coupled to High-Throughput Venomics for Snake Venom Characterisation with Parallel Bioactivity Assessment

Tanno Van Herpen, Radja Millenio Kosasih, Jeroen Kool * and Julien Slagboom *

This supplementary material contains 18 figures and 13 tables.

1. HIC method optimisation

The importance of an optimised HIC method is stressed by the fact that the resulting nanofractions are used to gather coagulation bioassay data and perform the HT venomics measurements, for which optimal HIC separations will provide the best eventual results. The HIC method used a 50 mM sodium phosphate buffer as mobile phase A (MP A) and 50 mM sodium phosphate buffer with the addition of 2M ammonium sulphate as mobile phase B (MP B). During the optimisation process, the venom of *Bothrops jararaca* was used to improve the separation. Below, the different gradients tested, one after the other, are shown in the figures presented. The last gradient shown in Figure S6 was slightly adjusted by extending the gradient from 5% mobile phase A to 75% from 40 minutes to 60 minutes, which visually gave the best results in terms of separation efficiency of most of the clearly visible peaks. This gradient was used for the remainder of this study as it was observed (data not shown) that for the other three venoms included in this study, also good separation results were achieved using this gradient.

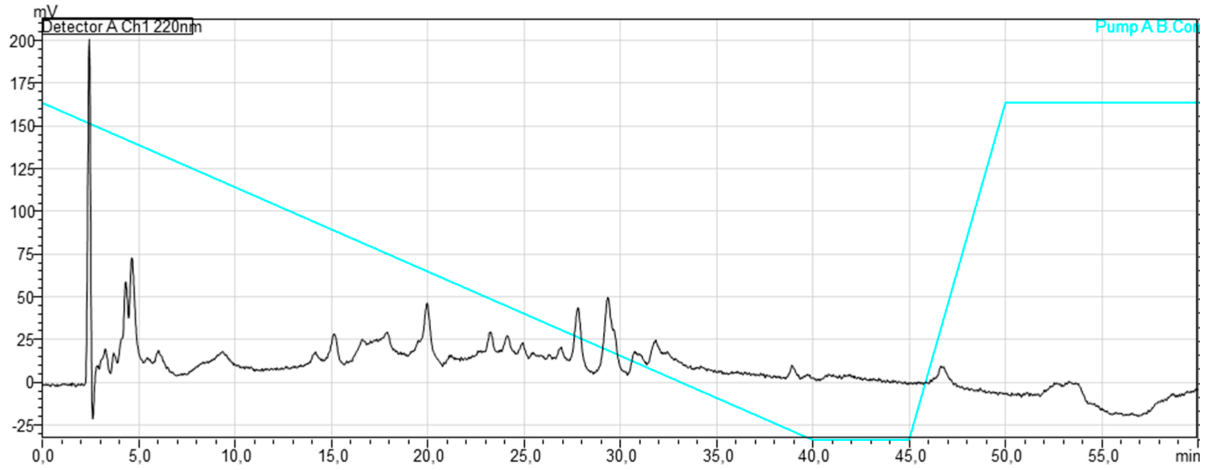

Figure S1. HIC LC-UV chromatogram of *Bothrops jararaca* venom analysed using the following gradient: The used gradient started at 20% mobile phase A, which was increased to 100% over a period of 40 minutes. It stayed for 5 minutes and after which the concentration was brought back to 20% in 5 minutes leading to a re-equilibration time of 10 minutes. On the right Y-axis, the concentration of mobile phase B during the gradient is depicted. The cyan line in the figure is a representation of the gradient during the gradient elution. The X-axis represents the retention time. On the left Y-axis the absorbance at 220 nm in mV corresponding to the black lined chromatogram is shown.

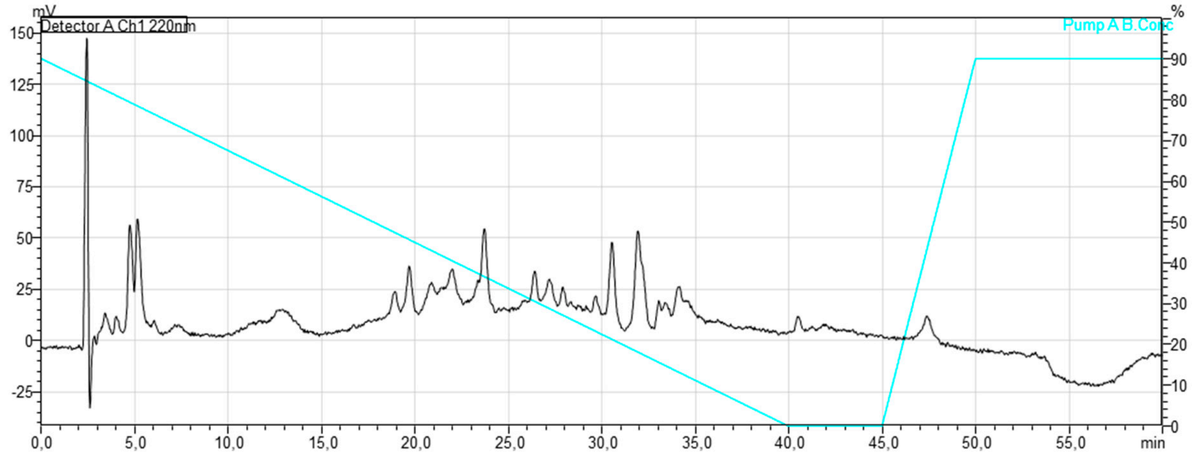

Figure S2. HIC LC-UV chromatogram of *Bothrops jararaca* venom analysed using the following gradient: The used gradient started at 10% mobile phase A, which was increased to 100% over a period of 40 minutes. It stayed for 5 minutes and after which the concentration was brought back to 10% in 5 minutes leading to a re-equilibration time of 10 minutes. On the right Y-axis, the concentration of mobile phase B during the gradient is depicted. The cyan line in the figure is a representation of the gradient during the gradient elution. The X-axis represents the retention time. On the left Y-axis the absorbance at 220 nm in mV corresponding to the black lined chromatogram is shown.

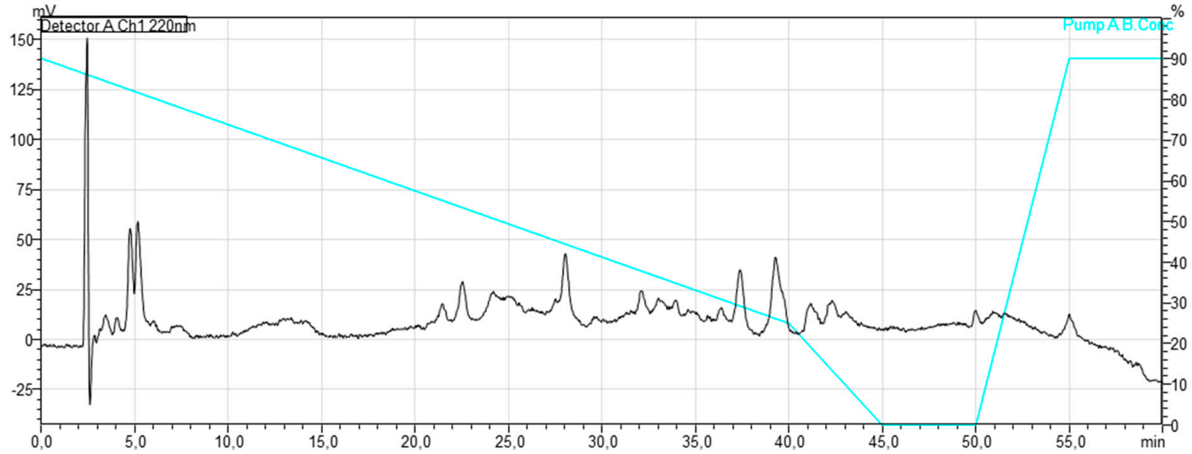

Figure S3. HIC LC-UV chromatogram of *Bothrops jararaca* venom analysed using the following gradient: The used gradient started at 10% mobile phase A, which was increased to 75% over a period of 40 minutes. Next, the concentration of mobile phase A was increased from 75% to 100% in 5 minutes, which marked the start of the washing step. After 5 minutes of washing the column on 100% A the concentration was brought back down to 5% A in a timespan of 5 minutes, where it stayed for 5 more minutes in order to re-equilibrate the column. On the right Y-axis, the concentration of mobile phase B during the gradient is depicted. The cyan line in the figure is a representation of the gradient during the gradient elution. The X-axis represents the retention time. On the left Y-axis the absorbance at 220 nm in mV corresponding to the black lined chromatogram is shown.

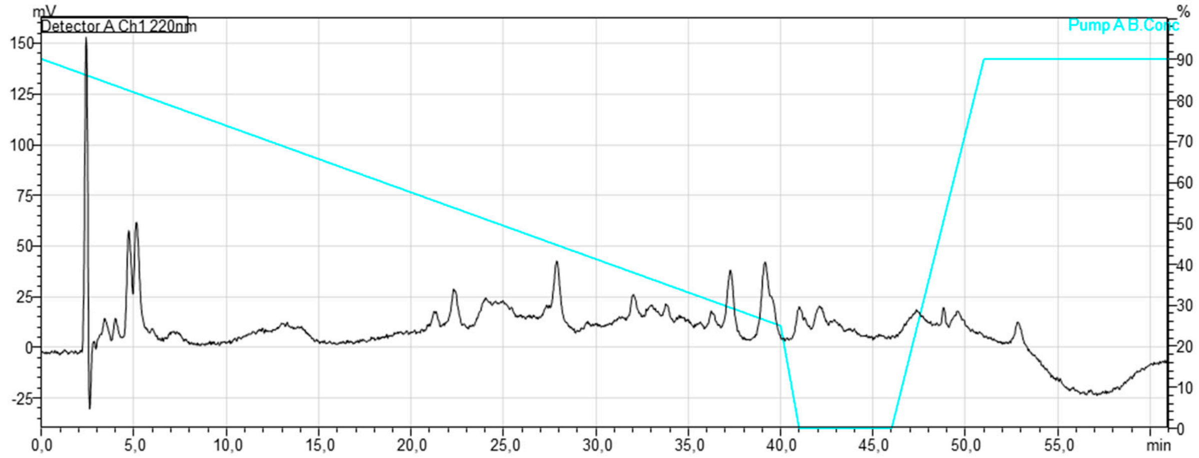

Figure S4. HIC LC-UV chromatogram of *Bothrops jararaca* venom analysed using the following gradient: The used gradient started at 10% mobile phase A, which was increased to 75% over a period of 40 minutes. Next, the concentration of mobile phase A was increased from 75% to 100% in 1 minute, which marked the start of the washing step. After 5 minutes of washing the column on 100% A the concentration was brought back down to 5% A in a timespan of 5 minutes, where it stayed for 10 more minutes in order to re-equilibrate the column. On the right Y-axis, the concentration of mobile phase B during the gradient is depicted. The cyan line in the figure is a representation of the gradient during the gradient elution. The X-axis represents the retention time. On the left Y-axis the absorbance at 220 nm in mV corresponding to the black lined chromatogram is shown.

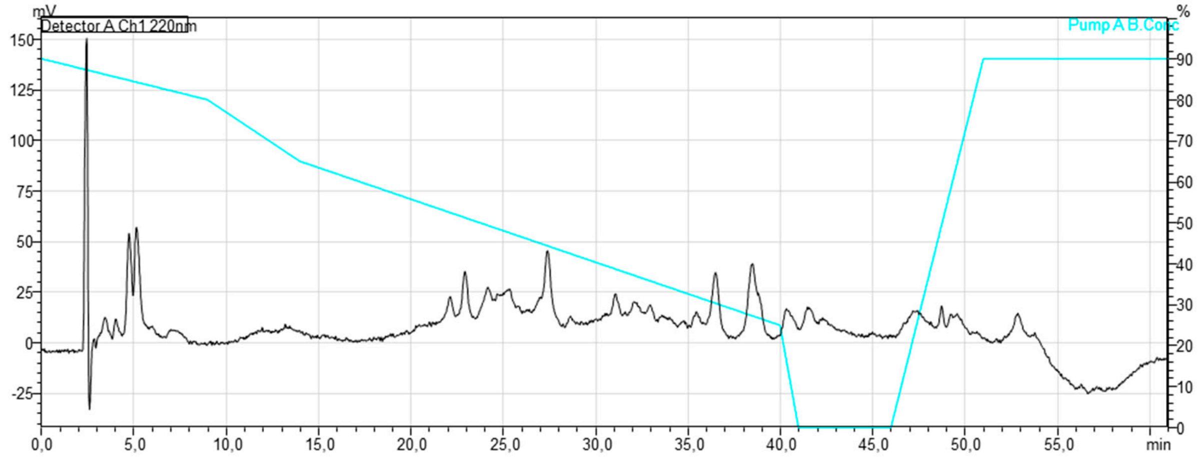

Figure S5. HIC LC-UV chromatogram of *Bothrops jararaca* venom analysed using the following gradient: The used gradient started at 10% mobile phase A, which was increased to 20% over a period of 9 minutes. Then the concentration went to 30% in 5 minutes. Next, the concentration of mobile phase A was first increased to 75% in 26 minutes after which it increased from 75% to 100% in 1 minute, which marked the start of the washing step. After 5 minutes of washing the column on 100% A the concentration was brought back down to 5% A in a timespan of 5 minutes, where it stayed for 10 more minutes in order to re-equilibrate the column. On the right Y-axis, the concentration of mobile phase B during the gradient is depicted. The cyan line in the figure is a representation of the gradient during the gradient elution. The X-axis represents the retention time. On the left Y-axis the absorbance at 220 nm in mV corresponding to the black lined chromatogram is shown.

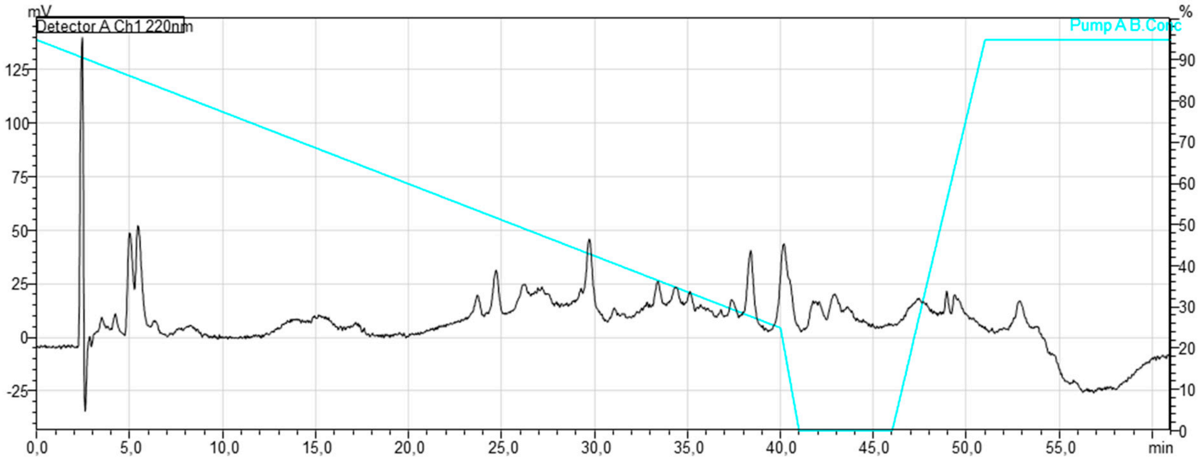

Figure S6. HIC LC-UV chromatogram of *Bothrops jararaca* venom analysed using the following gradient: The used gradient started at 5% mobile phase A, which was increased to 75% over a period of 40 minutes. Next, the concentration of mobile phase A was increased from 75% to 100% in 1 minute, which marked the start of the washing step. After 5 minutes of washing the column on 100% A the concentration was brought back down to 5% A in a timespan of 5 minutes, where it stayed for 10 more minutes in order to re-equilibrate the column. On the right Y-axis, the concentration of mobile phase B during the gradient is depicted. The cyan line in the figure is a representation of the gradient during the gradient elution. The X-axis represents the retention time. On the left Y-axis the absorbance at 220 nm in mV corresponding to the black lined chromatogram is shown.

2. Protein Score Chromatograms (PSCs) UniProt and transcriptomics databases

PSCs plot Mascot protein scores against fractionation retention time and are qualitative identification profiles. The additional transcriptomic searches use the databases designated *Bothrops asper* and *Bothrops jararaca* in the Methods; reference accessions do not independently establish species-specific toxin isoforms.

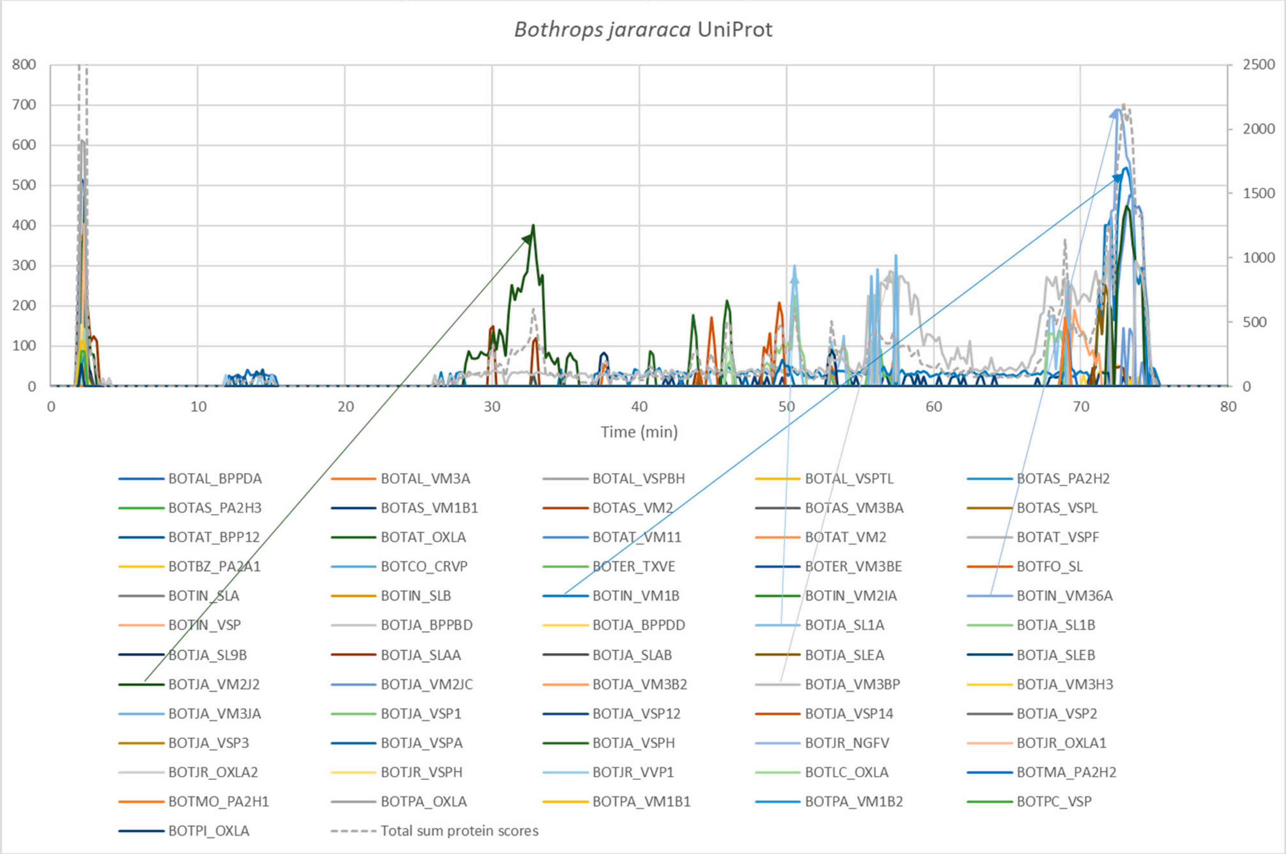

Figure S7. PSC of the *Bothrops jararaca* snake venom screened using the UniProt database with 5 selected protein matches connected to their corresponding graphs. The right y-axis shows the response of the total sum of all the protein scores.

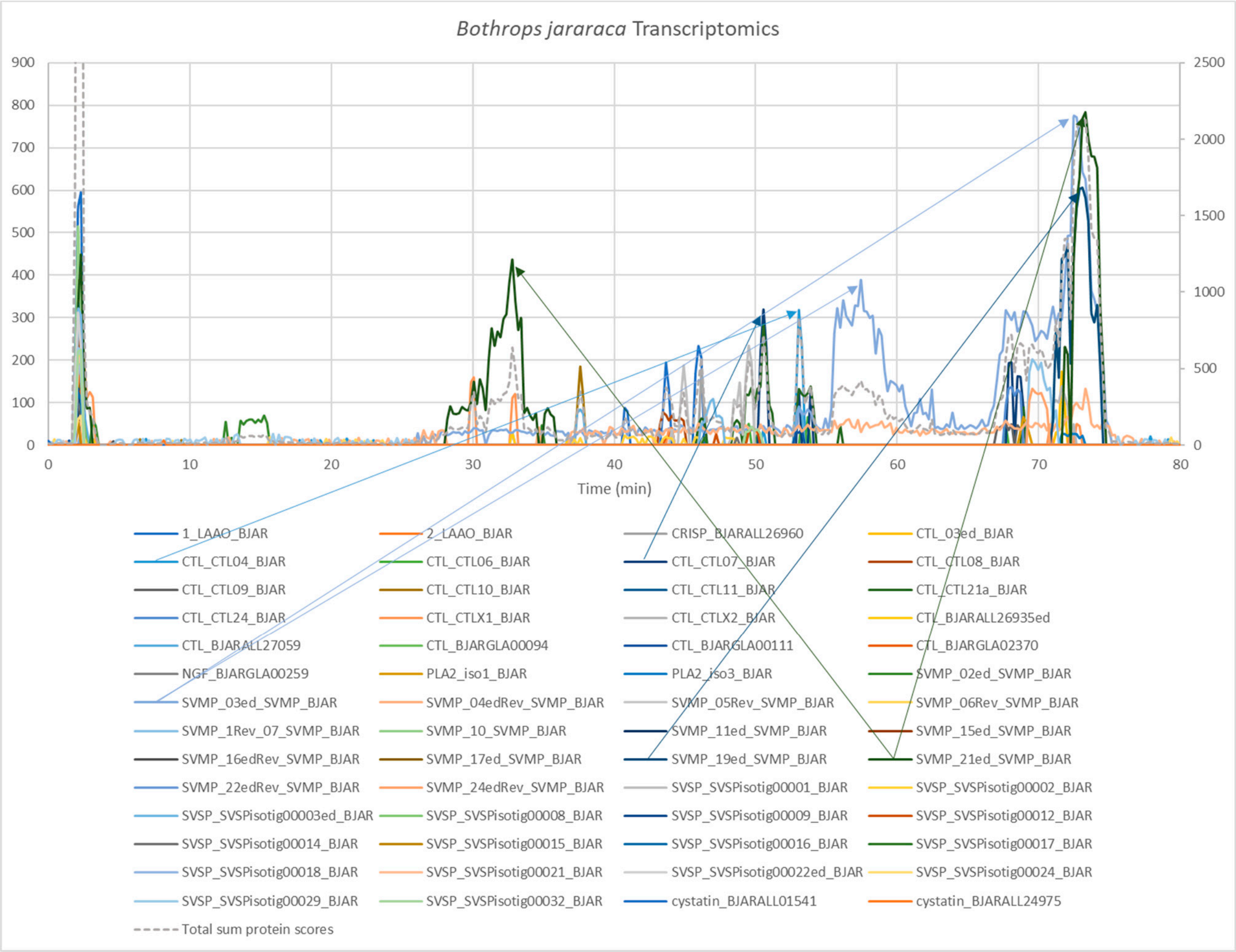

Figure S8. PSC of the *Bothrops jararaca* snake venom screened using the Transcriptomics database with 5 selected protein matches connected to their corresponding graphs. The right y-axis shows the response of the total sum of all the protein scores.

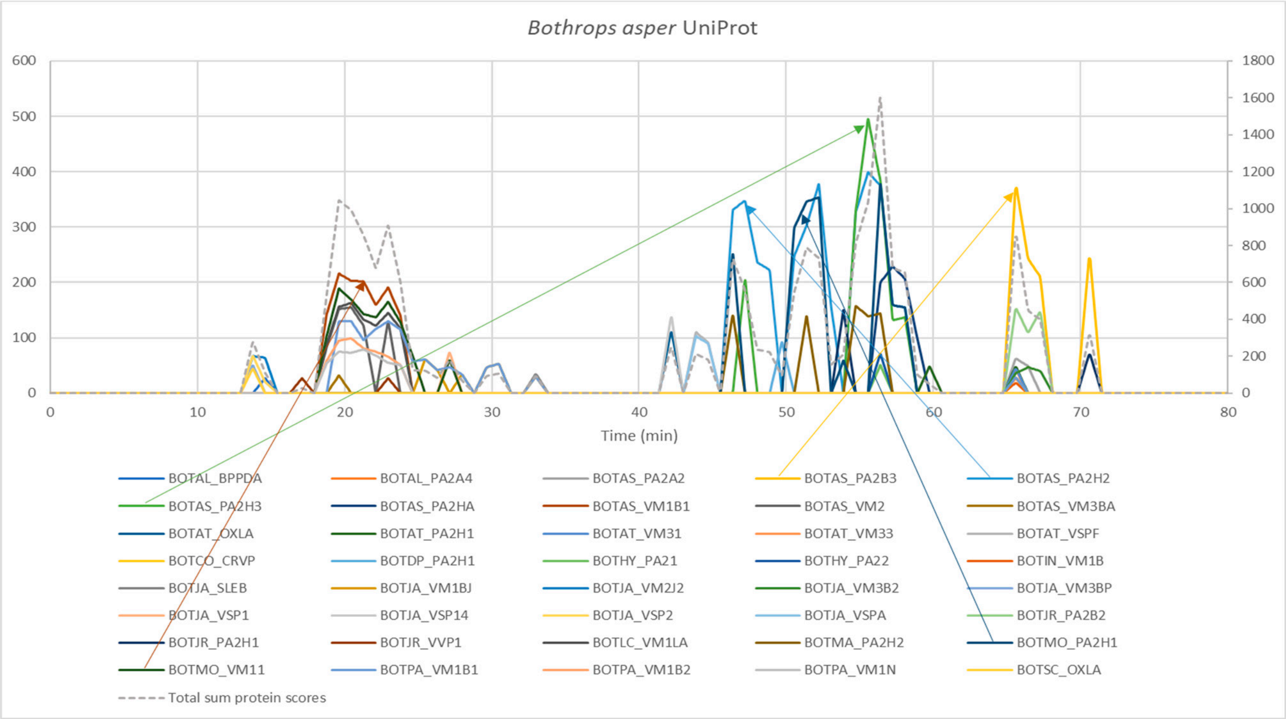

Figure S9. PSC of the *Bothrops asper* snake venom screened using the UniProt database with 5 selected protein matches connected to their corresponding graphs. The right y-axis shows the response of the total sum of all the protein scores.

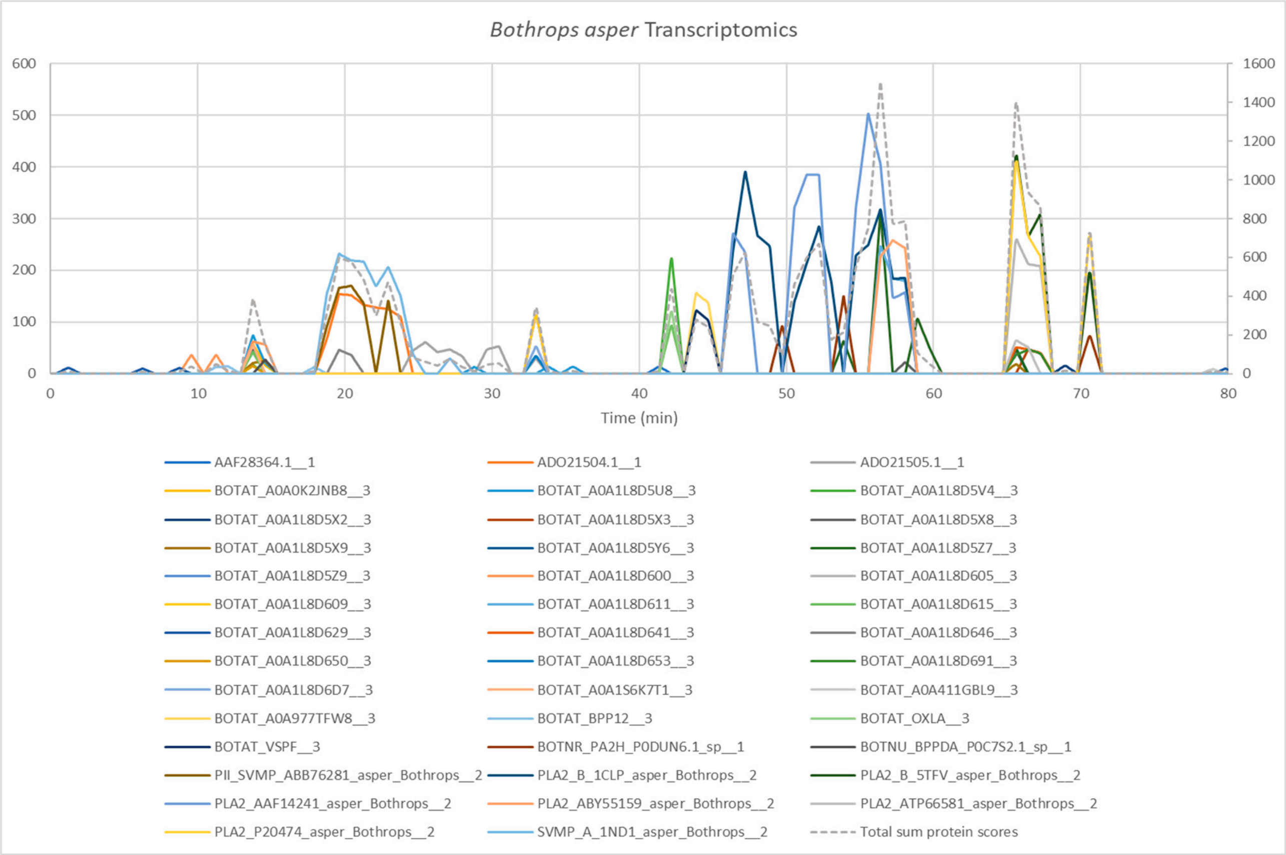

Figure S10. PSC of the *Bothrops asper* snake venom screened using the transcriptomics database with 5 selected protein matches connected to their corresponding graphs. The right y-axis shows the response of the total sum of all the protein scores.

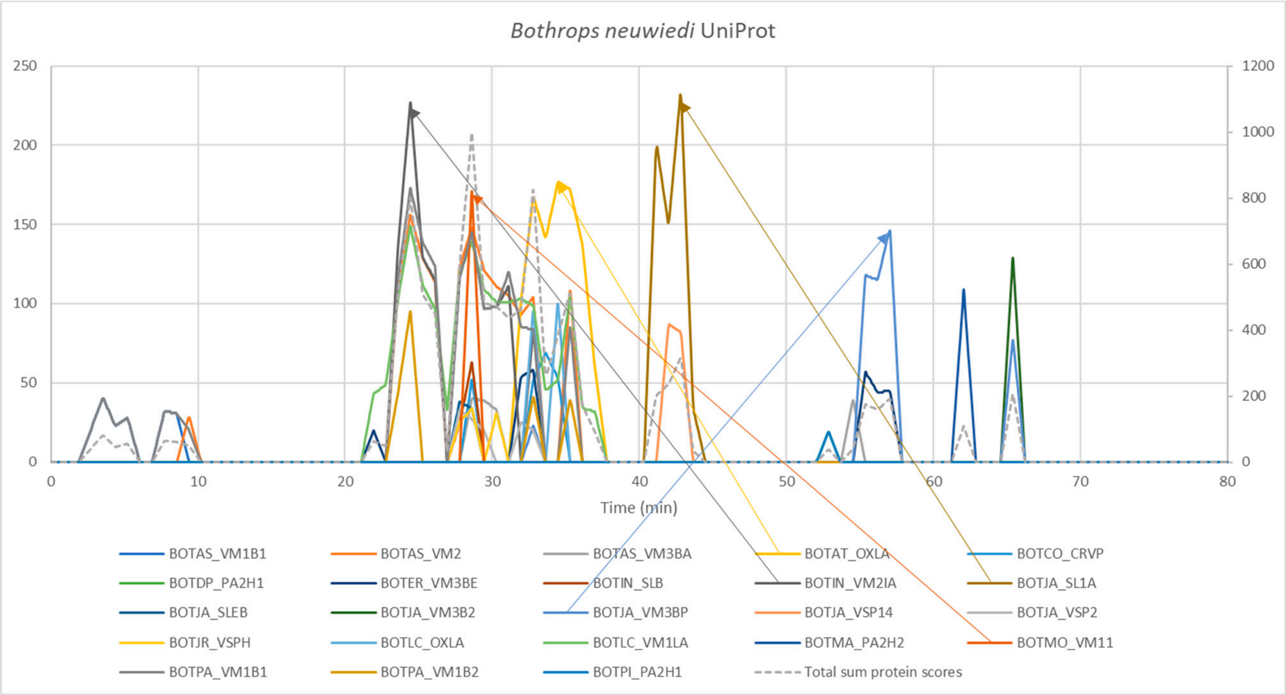

Figure S11. PSC of the *Bothrops neuwiedi* snake venom screened using the UniProt database with 5 selected protein matches connected to their corresponding graphs. The right y-axis shows the response of the total sum of all the protein scores.

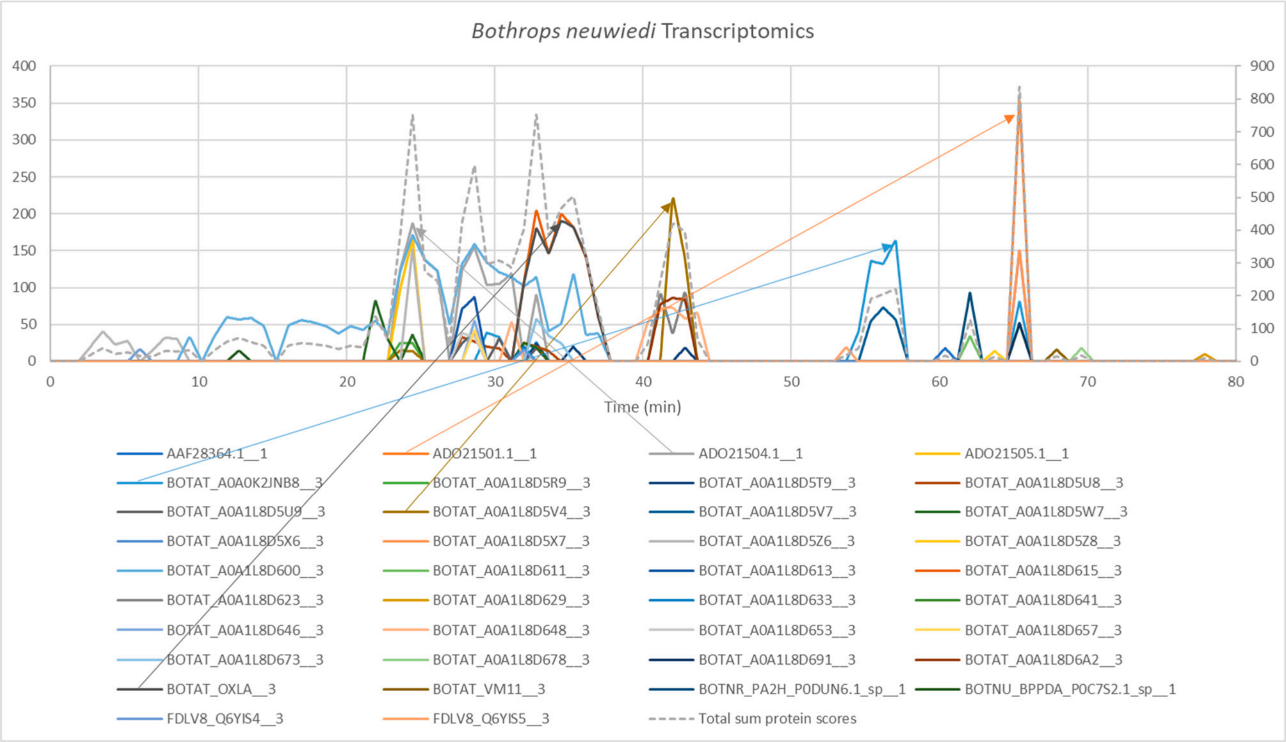

Figure S12. PSC of the *Bothrops neuwiedi* snake venom screened using the transcriptomics database with 5 selected protein matches connected to their corresponding graphs. The right y-axis shows the response of the total sum of all the protein scores.

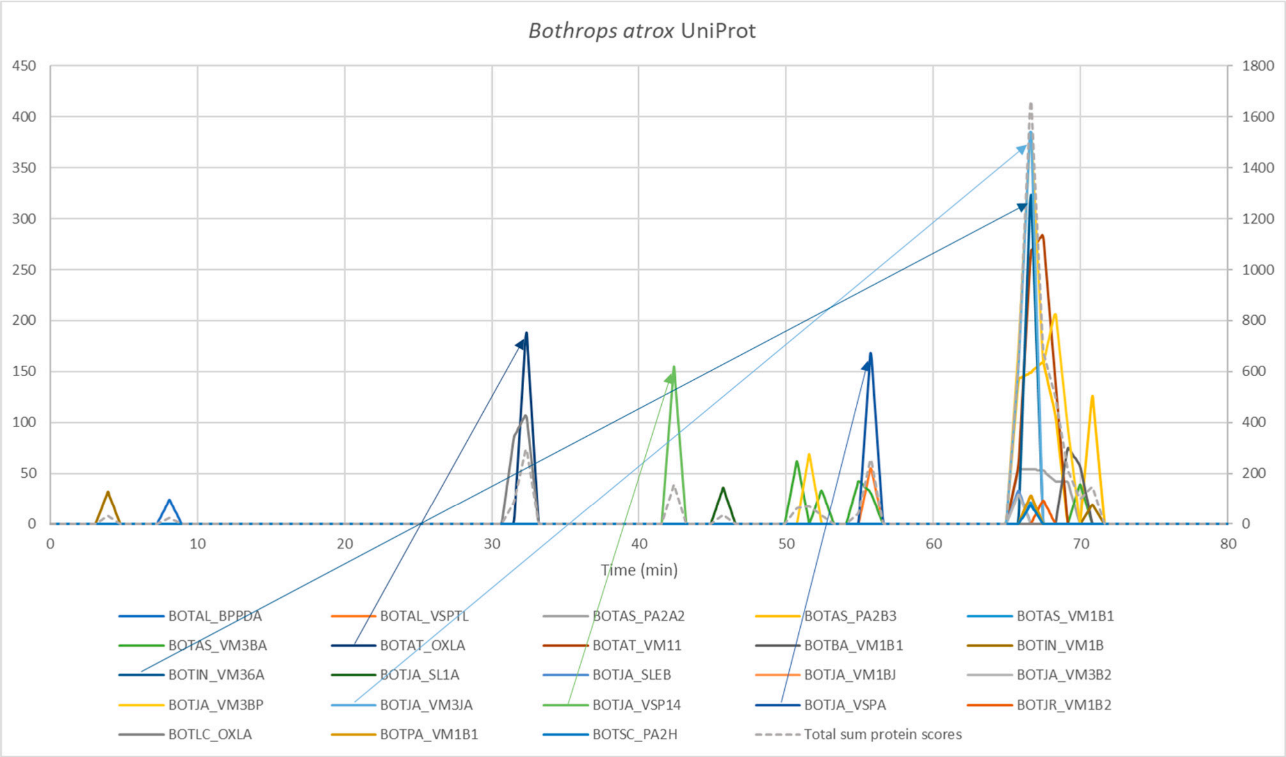

Figure S13. PSC of the *Bothrops atrox* snake venom screened using the UniProt database with 5 selected protein matches connected to their corresponding graphs. The right y-axis shows the response of the total sum of all the protein scores.

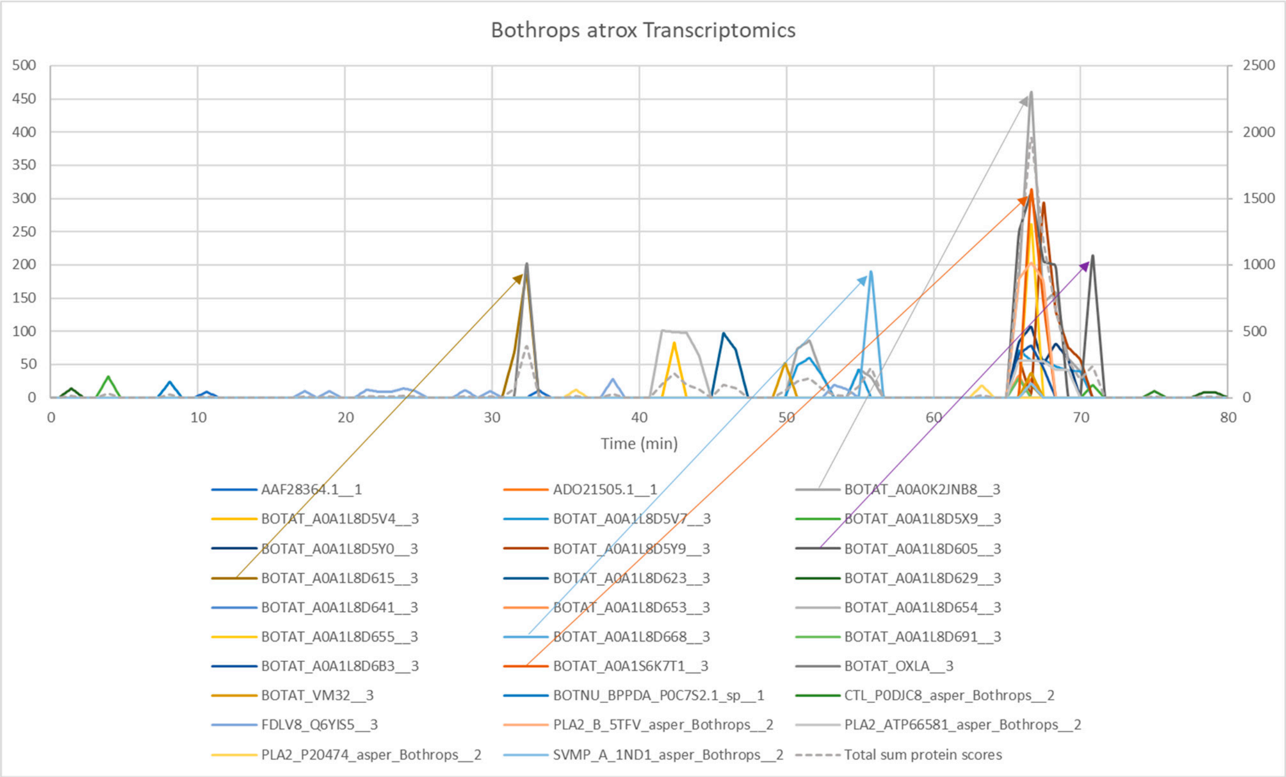

Figure S14. PSC of the *Bothrops atrox* snake venom screened using the transcriptomics database with 5 selected protein matches connected to their corresponding graphs. The right y-axis shows the response of the total sum of all the protein scores.

3. Transcriptomics LC-UV, Bioassay chromatograms, and PSCs.

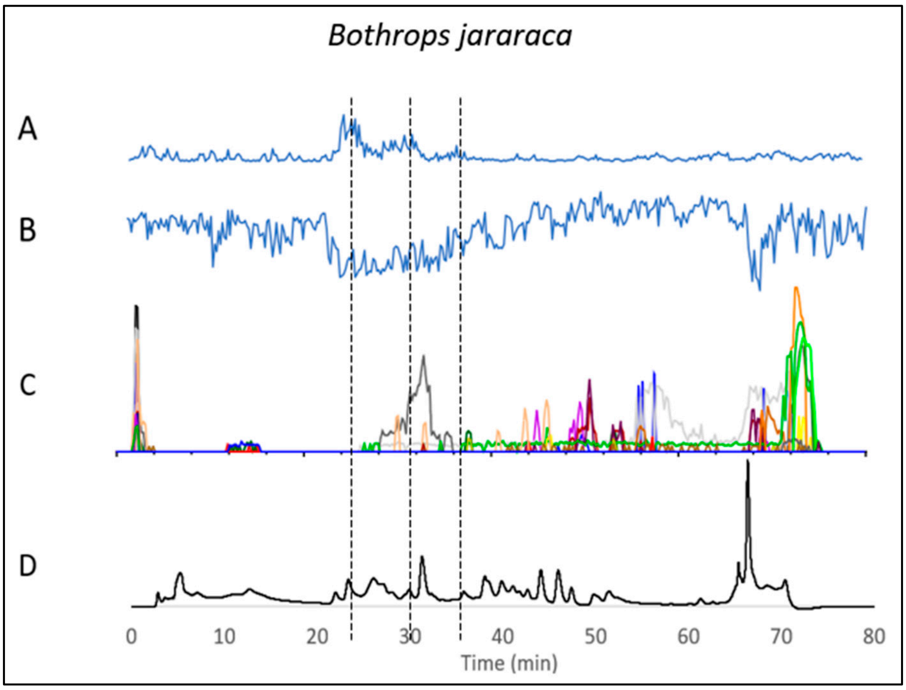

Figure S15. The searches were done using the Transcriptomics database. Protein Score Chromatograms (PSCs) of the *Bothrops jararaca* superimposed with its corresponding bioactivity and LC-UV chromatograms after separation using the optimised HIC method. For the results of *Bothrops jararaca* the 18 times dilution was used for cycle 5 and the 9 times dilution was used for cycle 80. A: Bioactivity chromatograms of cycle 5. B: Bioactivity chromatograms of cycle 80. C: The Protein Score Chromatograms. D: LC-UV chromatograms of the corresponding HIC-HPLC separations.

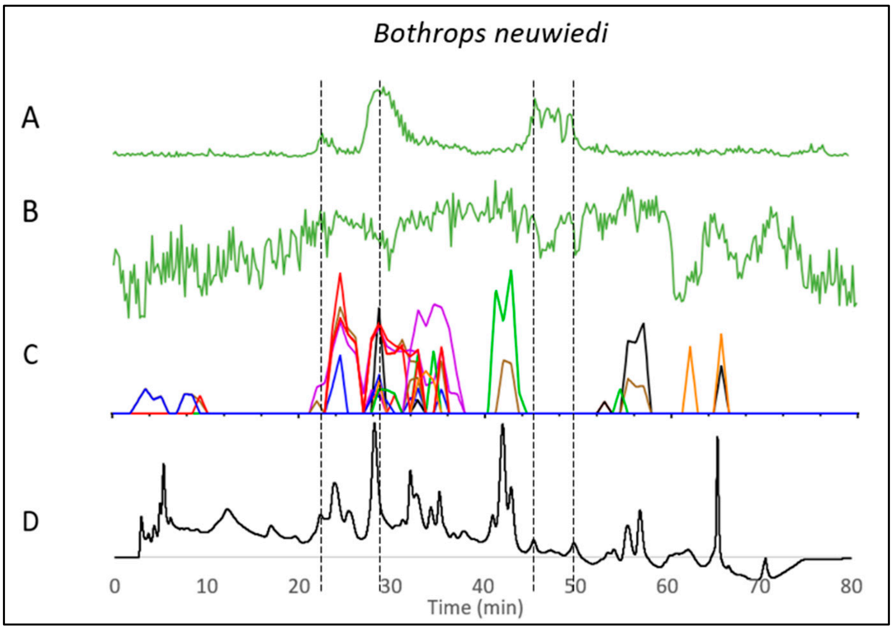

Figure S16. The searches were done using the Transcriptomics database. Protein Score Chromatograms (PSCs) of the *Bothrops neuwiedi* superimposed with its corresponding bioactivity and LC-UV chromatograms after separation using the optimised HIC method. For the results of the bioassays of the *Bothrops neuwiedi* the 36 times dilution for cycle 5 and the 18 times dilution for cycle 80 were processed. A: Bioactivity chromatograms of cycle 5. B: Bioactivity chromatograms of cycle 80. C: The Protein Score Chromatograms. D: LC-UV chromatograms of the corresponding HIC-HPLC separations.

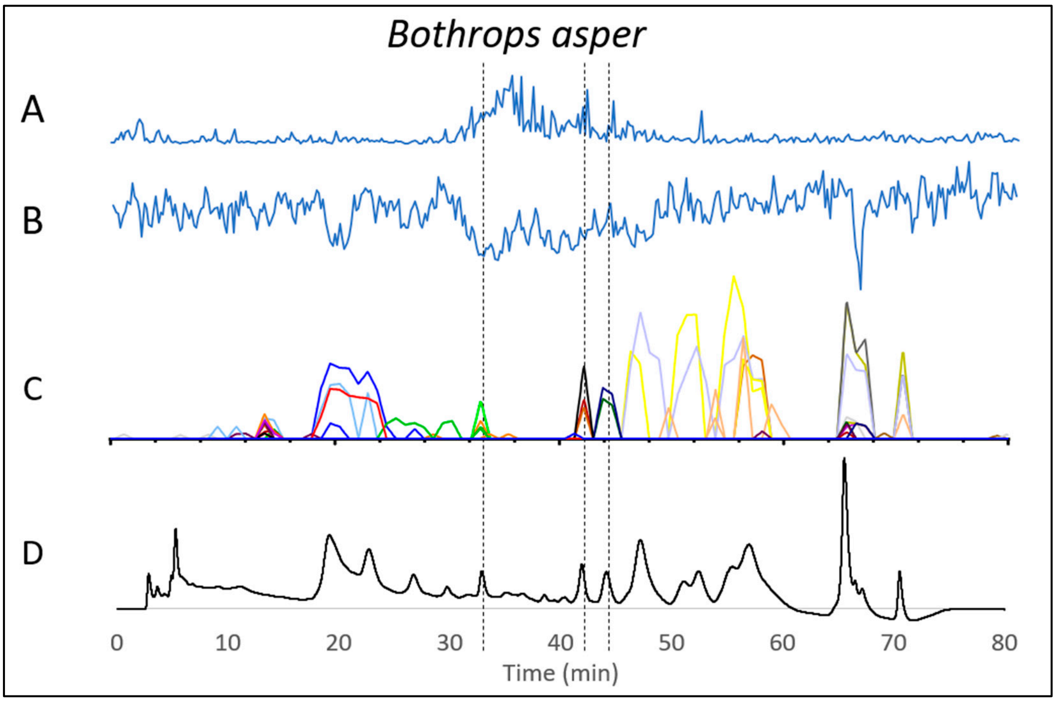

Figure S17. Transcriptomics database Protein Score Chromatograms (PSCs) of the *Bothrops asper* superimposed with the corresponding bioactivity and LC-UV chromatograms after separation using the optimised HIC method. For the results of the bioassays the 144 times dilution for cycle 5 and the 18 times dilution for cycle 80 were processed. A: Bioactivity chromatograms of cycle 5. B: Bioactivity chromatograms of cycle 80. C: The Protein Score Chromatograms. D: LC-UV chromatograms of the corresponding HIC-HPLC separations.

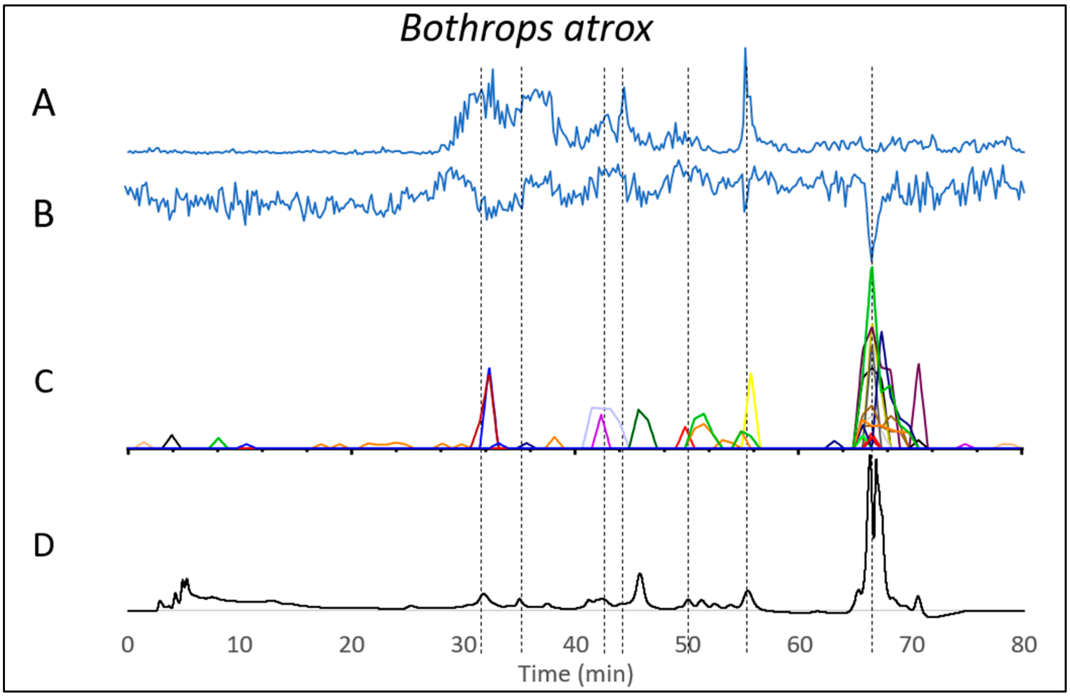

Figure S18. Transcriptomics database Protein Score Chromatograms (PSCs) of the *Bothrops atrox* superimposed with the corresponding bioactivity and LC-UV chromatograms after separation using the optimised HIC method. For the results of the bioassays the 144 times dilution for cycles 5 and 80 were processed. A: Bioactivity chromatograms of cycle 5. B: Bioactivity chromatograms of cycle 80. C: The Protein Score Chromatograms. D: LC-UV chromatograms of the corresponding HIC-HPLC separations.

4. Nomenclature UniProt proteomics results

Table S1. Nomenclature of all proteins found in *Bothrops asper* venom using the UniProt database and VenomZone from the Swiss-Prot group of the SIB Swiss Institute of Bioinformatics.

Bothrops asper HIC			
Protein ID	Protein Description	Family	Subfamily, Sub-Subfamily
BOTAS_VM1B1	Snake venom metalloproteinase BaP1	SVMP	P-I
BOTAS_VM2	(Zinc) Metalloproteinase/Disintegrin	SVMP	P-II, P-IId
BOTIN_VM1B	Snake venom metalloproteinase BITM02A	SVMP	P-I
BOTAS_VM3BA	Snake venom metalloproteinase basparin-A	SVMP	P-III, P-IIIa
BOTJA_VM1BJ	Snake venom metalloproteinase bothrojaractivase	SVMP	P-I
BOTAT_VM31	Zinc metalloproteinase-disintegrin-like batroxstatin-1	SVMP	P-III, P-IIIa
BOTAT_VM33	Zinc metalloproteinase-disintegrin-like batroxstatin-3	SVMP	P-III, P-IIIa
BOTJA_VM2J2	Snake venom metalloproteinase jararafibrase-2	SVMP	P-II, P-Iia
BOTJA_VM3B2	Zinc metalloproteinase-disintegrin-like bothrojarin-2	SVMP	P-III, P-IIIa
BOTJA_VM3BP	Zinc metalloproteinase-disintegrin-like bothropasin	SVMP	P-III, P-IIIb
BOTLC_VM1LA	Snake venom metalloproteinase leucurolysin-A	SVMP	P-I
BOTMO_VM11	Snake venom metalloproteinase BmooMP-I	SVMP	P-I
BOTPA_VM1B1	Snake venom metalloproteinase BnP1	SVMP	P-I
BOTPA_VM1B2	Snake venom metalloproteinase BnP2	SVMP	P-I
BOTPA_VM1N	Snake venom metalloproteinase neuwiedase	SVMP	P-I
BOTAL_PA2A4	Acidic phospholipase A_2 SpII RP4	PLA_2	Group 2, D49
BOTAS_PA2A2	Acidic phospholipase A_2 2	PLA_2	Group 2, D49
BOTHY_PA22	Basic myotoxic phospholipase A_2 PhTX-II	PLA_2	Group 2, D49

Bothrops asper HIC			
Protein ID	Protein Description	Family	Subfamily, Sub-Subfamily
BOTAS_PA2B3	Basic phospholipase A_2 myotoxin I	PLA_2	Group 2, D49
BOTAS_PA2H2	Basic phospholipase A_2 homolog 2	PLA_2	Group 2, K49
BOTAS_PA2H3	Basic phospholipase A_2 homolog M1-3-3	PLA_2	Group 2, K49
BOTAS_PA2HA	Basic phospholipase A_2 homolog 4a	PLA_2	Group 2, K49
BOTDP_PA2H1	Basic phospholipase A_2 homolog BdipTx-I	PLA_2	Group 2, K49
BOTHY_PA21	Basic phospholipase A_2 Ph-TX1	PLA_2	Group 2, D49
BOTJR_PA2B2	Basic phospholipase A_2 homolog bothropstoxin-II	PLA_2	Group 2, D49
BOTJR_PA2H1	Basic phospholipase A_2 homolog bothropstoxin-I	PLA_2	Group 2, K49
BOTMA_PA2H2	Basic phospholipase A_2 homolog BmajPLA2-II	PLA_2	Group 2, K49
BOTMO_PA2H1	Basic phospholipase A_2 homolog MjTX-I	PLA_2	Group 2, K49
BOTAL_BPPDA	Bradykinin-potentiating peptide 13a	BPP	
BOTCO_CRVP	Cysteine-rich venom protein Bco13	CRISP-V	
BOTAT_OXLA	L-amino-acid oxidase	LAO	FIG1
BOTSC_OXLA	L-amino-acid oxidase	LAO	FIG1
BOTAT_PA2H1	Phospholipase A_2 homolog 1	PLA_2	Group 2, K49
BOTJA_VSP1	Platelet-aggregating proteinase PA-BJ	SVSP	Peptidase S1
BOTJA_VSP14	Snake venom serine protease HS114	SVSP	Peptidase S1
BOTJA_SLEB	Snaclec botrocetin subunit beta	Snaclec	
BOTJA_VSPA	Thrombin-like enzyme bothrombin	SVSP	Peptidase S1
BOTAT_VSPF	Thrombin-like enzyme batroxobin	SVSP	Peptidase S1
BOTJA_VSP2	Thrombin-like enzyme KN-BJ 2	SVSP	Peptidase S1
BOTJR_VVP1	Venom vasodilator peptide	No family	

Table S2. Nomenclature of all toxins (and other proteins) found in *Bothrops atrox* snake venom using the UniProt database and VenomZone from the Swiss-Prot group of the SIB Swiss Institute of Bioinformatics.

Bothrops atrox HIC			
Protein ID	Protein Description	Family	Subfamily, Sub-Subfamily
BOTAL_BPPDA	Bradykinin-potentiating peptide 13a	BPP	
BOTAL_VSPTL	Snake venom serine protease BthaTL	SVSP	Peptidase S1
BOTAS_PA2A2	Acidic phospholipase A_2 2	PLA_2	Group 2, D49
BOTAS_PA2B3	Basic phospholipase A_2 myotoxin I	PLA_2	Group 2, D49
BOTAS_VM1B1	Snake venom metalloproteinase BaP1	SVMP	P-I
BOTAS_VM3BA	Snake venom metalloproteinase basparin-A	SVMP	P-III, P-IIIa
BOTAT_OXLA	L-amino-acid oxidase	LAO	FIG1
BOTAT_VM11	Snake venom metalloproteinase atroxlysin-1	SVMP	P-I
BOTBA_VM1B1	Zinc metalloproteinase barnettlysin-1	SVMP	P-I
BOTIN_VM1B	Snake venom metalloproteinase BITM02A	SVMP	P-I
BOTIN_VM36A	Zinc metalloproteinase-disintegrin-like BITM06A	SVMP	P-III, P-IIIa
BOTJA_SL1A	Snaclec GPIB-binding protein subunit alpha	Snaclec	
BOTJA_SLEB	Snaclec botrocetin subunit beta	Snaclec	
BOTJA_VM1BJ	Snake venom metalloproteinase bothrojaractivase	SVMP	P-I
BOTJA_VM3B2	Zinc metalloproteinase-disintegrin-like bothrojarin-2	SVMP	P-III, P-IIIa
BOTJA_VM3BP	Zinc metalloproteinase-disintegrin-like bothropasin	SVMP	P-III, P-IIIb
BOTJA_VM3JA	Zinc metalloproteinase-disintegrin-like jararhagin	SVMP	P-III, P-IIIb

Bothrops atrox HIC			
Protein ID	Protein Description	Family	Subfamily, Sub-Subfamily
BOTJA_VSP14	Snake venom serine protease HS114	SVSP	Peptidase S1
BOTJA_VSPA	Thrombin-like enzyme bothrombin	SVSP	Peptidase S1
BOTJR_VM1B2	Snake venom metalloproteinase BjussuMP-2	SVMP	P-I
BOTLC_OXLA	L-amino-acid oxidase	LAO	FIG1
BOTPA_VM1B1	Snake venom metalloproteinase BnP1	SVMP	P-I
BOTSC_PA2H	Basic phospholipase A_2 homolog Bsc-K49	PLA_2	Group 2, K49

Table S3. Nomenclature of all toxins (and other proteins) found in *Bothrops neuwiedi* snake venom using the UniProt database and VenomZone from the Swiss-Prot group of the SIB Swiss Institute of Bioinformatics.

Bothrops neuwiedi HIC			
Protein ID	Protein Description	Family	Subfamily, Sub-Subfamily
BOTAS_VM1B1	Snake venom metalloproteinase BaP1	SVMP	P-I
BOTAS_VM2	(Zinc) Metalloproteinase/Disintegrin	SVMP	P-II, P-IId
BOTAS_VM3BA	Snake venom metalloproteinase basparin-A	SVMP	P-III, P-IIIa
BOTAT_OXLA	L-amino-acid oxidase	LAO	FIG1
BOTCO_CRVP	Cysteine-rich venom protein Bco13	CRISP-V	
BOTDP_PA2H1	Basic phospholipase A_2 homolog BdipTx-I	PLA_2	Group 2, K49
BOTER_VM3BE	Zinc metalloproteinase-disintegrin-like berythractivase	SVMP	P-III, P-IIIa
BOTIN_SLB	Snaclec bothroinsularin subunit beta	Snaclec	
BOTIN_VM2IA	Snake venom metalloproteinase insularinase-A and dis-integrin insularin	SVMP	P-II, P-IIa
BOTJA_SL1A	Snaclec GPIB-binding protein subunit alpha	Snaclec	
BOTJA_SLEB	Snaclec botrocetin subunit beta	Snaclec	
BOTJA_VM3B2	Zinc metalloproteinase-disintegrin-like bothrojarin-2	SVMP	P-III, P-IIIa
BOTJA_VM3BP	Zinc metalloproteinase-disintegrin-like bothropasin	SVMP	P-III, P-IIIb
BOTJA_VSP14	Snake venom serine protease HS114	SVSP	Peptidase S1
BOTJA_VSP2	Thrombin-like enzyme KN-BJ 2	SVSP	Peptidase S1
BOTJR_VSPH	Snake venom serine protease homolog	SVSP	Peptidase S1
BOTLC_OXLA	L-amino-acid oxidase	LAO	FIG1
BOTLC_VM1LA	Snake venom metalloproteinase leucurolysin-A	SVMP	P-I
BOTMA_PA2H2	Basic phospholipase A_2 homolog BmajPLA2-II	PLA_2	Group 2, K49
BOTMO_VM11	Snake venom metalloproteinase BmooMP-I	SVMP	P-I
BOTPA_VM1B1	Snake venom metalloproteinase BnP1	SVMP	P-I
BOTPA_VM1B2	Snake venom metalloproteinase BnP2	SVMP	P-I
BOTPI_PA2H1	Basic phospholipase A_2 homolog piratoxin-1	PLA_2	Group 2, K49

Table S4. Nomenclature of all toxins (and other proteins) found in *Bothrops jararaca* snake venom using the UniProt database and VenomZone from the Swiss-Prot group of the SIB Swiss Institute of Bioinformatics.

Bothrops jararaca HIC			
Protein ID	Protein Description	Family	Subfamily, Sub-Subfamily
BOTAL_BPPDA	Bradykinin-potentiating peptide 13a	BPP	
BOTAL_VM3A	Zinc metalloproteinase-disintegrin-like alternagin	SVMP	P-III, P-IIIb
BOTAL_VSPBH	Thrombin-like enzyme bhalternin	SVSP	Peptidase S1
BOTAL_VSPTL	Snake venom serine protease BthaTL	SVSP	Peptidase S1
BOTAS_PA2H2	Basic phospholipase A_2 homolog 2	PLA_2	Group 2, K49
BOTAS_PA2H3	Basic phospholipase A_2 homolog M1-3-3	PLA_2	Group 2, K49
BOTAS_VM1B1	Snake venom metalloproteinase BaP1	SVMP	P-I
BOTAS_VM2	(Zinc) Metalloproteinase/Disintegrin	SVMP	P-II, P-IId
BOTAS_VM3BA	Snake venom metalloproteinase basparin-A	SVMP	P-III, P-IIIa
BOTAS_VSPL	Thrombin-like enzyme asperase	SVSP	Peptidase S1
BOTAT_BPP12	Bradykinin-potentiating peptide 12a	BPP	
BOTAT_OXLA	L-amino-acid oxidase	LAO	FIG1
BOTAT_VM11	Snake venom metalloproteinase atroxlysin-1	SVMP	P-I
BOTAT_VM2	(Zinc) Metalloproteinase/Disintegrin	SVMP	P-II, P-IId
BOTAT_VSPF	Thrombin-like enzyme batroxobin	SVSP	Peptidase S1
BOTBZ_PA2A1	Acidic phospholipase A_2 braziliase-I	PLA_2	Group 2, D49
BOTCO_CRVP	Cysteine-rich venom protein Bco13	CRISP-V	
BOTER_TXVE	Snake venom vascular endothelial growth factor toxin	PDGF/VEGF	VEGF
BOTER_VM3BE	Zinc metalloproteinase-disintegrin-like berythractivase	SVMP	P-III, P-IIIa
BOTFO_SL	Snaclec Bfon18	Snaclec	
BOTIN_SLA	Snaclec bothroinsularin subunit alpha	Snaclec	
BOTIN_SLB	Snaclec bothroinsularin subunit beta	Snaclec	
BOTIN_VM1B	Snake venom metalloproteinase BITM02A	SVMP	P-I
BOTIN_VM2IA	Snake venom metalloproteinase insularinase-A/Disintegrin insularin	SVMP	P-II, P-IIa
BOTIN_VM36A	Zinc metalloproteinase-disintegrin-like BITM06A	SVMP	P-III, P-IIIa
BOTIN_VSP	Snake venom serine protease BITS01A	SVSP	Peptidase S1
BOTJA_BPPBD	Bradykinin-potentiating peptide 11d	BPP	
BOTJA_BPPDD	Bradykinin-potentiating peptide 13d	BPP	
BOTJA_SL1A	Snaclec GPIB-binding protein subunit alpha	Snaclec	
BOTJA_SL1B	Snaclec GPIB-binding protein subunit beta	Snaclec	
BOTJA_SL9B	Coagulation factor IX/factor X-binding protein subunit B	Snaclec	
BOTJA_SLAA	Snaclec bothrojaracin subunit alpha	Snaclec	
BOTJA_SLAB	Snaclec bothrojaracin subunit beta	Snaclec	
BOTJA_SLEA	Snaclec botrocetin subunit alpha	Snaclec	
BOTJA_SLEB	Snaclec botrocetin subunit beta	Snaclec	
BOTJA_VM2J2	Snake venom metalloproteinase jararafibrase-2	SVMP	P-II, P-IIa
BOTJA_VM2JC	Zinc metalloproteinase/disintegrin	SVMP	P-II, P-IIa
BOTJA_VM3B2	Zinc metalloproteinase-disintegrin-like bothropasin	SVMP	P-III, P-IIIa
BOTJA_VM3BP	Zinc metalloproteinase-disintegrin-like bothropasin	SVMP	P-III, P-IIIb
BOTJA_VM3H3	Zinc metalloproteinase-disintegrin-like HF3	SVMP	P-III, P-IIIa
BOTJA_VM3JA	Zinc metalloproteinase-disintegrin-like jararhagin	SVMP	P-III, P-IIIb

Bothrops jararaca HIC			
Protein ID	Protein Description	Family	Subfamily, Sub-Subfamily
BOTJA_VSP1	Platelet-aggregating proteinase PA-BJ	SVSP	Peptidase S1
BOTJA_VSP12	Snake venom serine protease HS112	SVSP	Peptidase S1
BOTJA_VSP14	Snake venom serine protease HS114	SVSP	Peptidase S1
BOTJA_VSP2	Thrombin-like enzyme KN-BJ 2	SVSP	Peptidase S1
BOTJA_VSP3	Snake venom serine protease BPA	SVSP	Peptidase S1
BOTJA_VSPA	Thrombin-like enzyme bothrombin	SVSP	Peptidase S1
BOTJA_VSPH	Snake venom serine protease homolog HS120	SVSP	Peptidase S1
BOTJR_NGFV	Venom nerve growth factor	NGF-beta	
BOTJR_OXLA1	L-amino-acid oxidase BjussuLAAO-I	LAO	FIG1
BOTJR_OXLA2	L-amino-acid oxidase BjussuLAAO-II	LAO	FIG1
BOTJR_VSPH	Snake venom serine protease homolog	SVSP	Peptidase S1
BOTJR_VVP1	Venom vasodilator peptide	No family	
BOTLC_OXLA	L-amino-acid oxidase	LAO	FIG1
BOTMA_PA2H2	Basic phospholipase A_2 homolog BmajPLA2-II	PLA_2	Group 2, K49
BOTMO_PA2H1	Basic phospholipase A_2 homolog MjTX-I	PLA_2	Group 2, K49
BOTPA_OXLA	L-amino-acid oxidase	LAO	FIG1
BOTPA_VM1B1	Snake venom metalloproteinase BnP1	SVMP	P-I
BOTPA_VM1B2	Snake venom metalloproteinase BjussuMP-2	SVMP	P-I
BOTPC_VSP	Snake venom serine protease pictobin	SVSP	Peptidase S1
BOTPI_OXLA	L-amino-acid oxidase	LAO	FIG1

5. HT venomics PSC data correlation with bioactivity peaks

The activities in Tables S5–S13 refer to the measured fractions. Database annotations and co-eluting candidate sequence matches do not establish the contribution of an individual toxin to that response. Further separation and functional testing would be required to confirm individual toxin–activity assignments.

Table S5. An overview of the coagulopathy profile characteristics of the *Bothrops snake* venoms analysed using the main timepoints where effects in the coagulation bioassay were observed. Pro: Procoagulation effect. Anti: Anticoagulation effect.

Coagulopathy characteristics					
Viperidae					
Species	Timepoint (min)	Effect	Species	Timepoint (min)	Effect
Bothrops asper	20	Anti	*Bothrops jararaca*	24	Pro
	33	Pro		30	Pro
	42	Pro		35	Pro
	45	Pro		66	Anti
	66	Anti			
Bothrops atrox	32	Pro	*Bothrops neuwiedi*	24	Pro
	36	Pro		28	Pro
	42	Pro		46	Pro
	50	Pro		50	Pro
	55	Pro		62	Anti
	66	Anti		66	Anti

6. UniProt bioactivity references

Table S6. Overview of the tentatively identified bioactive coagulopathic toxins in *Bothrops jararaca* venom by correlating coagulation bioassay peaks with HT venomics PSC data retrieved from database searches using the UniProt database and VenomZone from the Swiss-Prot group of the SIB Swiss Institute of Bioinformatics. PAIT = Platelet aggregation inhibition toxin. HIT = Hemostasis impairing toxin. ICIT = Ion channel impairing toxin.

Bothrops jararaca					
Bioassay peak timepoint (Min)	Observed effect	Protein ID	Protein family	Subfamily, Sub-Subfamily	Theoretical effect(s)
23	Procoagulant	-	-	-	-
30	Procoagulant	BOTJA_VM2J2	SVMP	P-II, P-IIa	PAIT
36	Procoagulant	BOTFO_SL	Snaclec		HIT
		BOTJA_VM3BP	SVMP	P-III, P-IIIb	PAIT, ICIT, HIT
66	Anticoagulant	BOTJA_VM3BP	SVMP	P-III, P-IIIb	PAIT, ICIT, HIT
		BOTJA_SL1A	Snaclec		PAIT, HIT
		BOTJA_SL1B	Snaclec		PAIT, HIT

Table S7. Overview of the tentatively identified bioactive coagulopathic toxins in *Bothrops neuwiedi* venom by correlating coagulation bioassay peaks with HT venomics PSC data retrieved from database searches using the UniProt database and VenomZone from the Swiss-Prot group of the SIB Swiss Institute of Bioinformatics. Platelet aggregation inhibition toxin: PAIT, Hemostasis impairing toxin: HIT, Ion channel impairing toxin: ICIT, Blood coagulation cascade activation: BCCA, Fibrinogenolytic activity: FA, Platelet aggregation activating toxin: PAAT, Blood coagulation cascade inhibition: BCCI, Hydrolase: HYD.

Bothrops neuwiedi					
Bioassay peak timepoint (Min)	Observed effect	Protein ID	Family	Subfamily, Sub-Subfamily	Theoretical effect(s)
24	Procoagulant	BOTIN_VM2IA	SVMP	P-II, P-IIa	BCCA, PAIT, HIT
		BOTAS_VM2	SVMP	P-II, P-IId	PAIT
		BOTLC_VM1LA	SVMP	P-I	PAIT
		BOTPA_VM1B1	SVMP	P-I	FA
		BOTPA_VM1B2	SVMP	P-I	FA
28	Procoagulant	BOTCO_CRVP	CRISP-V		ICIT
		BOTAS_VM3BA	SVMP	P-III, P-IIIa	BCCA, FA
		BOTJA_VSP2	SVSP	Peptidase S1	BCCA
		BOTJR_VSPH	SVSP	Peptidase S1	HIT
		BOTJA_SLEB	Snaclec		PAAT
		BOTAS_VM2	SVMP	P-II, P-IId	PAIT
		BOTMO_VM11	SVMP	P-I	PAAT
		BOTIN_SLB	Snaclec		PAIT, BCCI
		BOTLC_VM1LA	SVMP	P-I	PAIT
		BOTPA_VM1B1	SVMP	P-I	FA
46	Procoagulant	-	-	-	-
50	Procoagulant	-	-	-	-
62	Anticoagulant	BOTMA_PA2H2	PLA_2	Group 2, K49	HYD
66	Anticoagulant	-	-	-	-

Table S8. Overview of the tentatively identified bioactive coagulopathic toxins in *Bothrops asper* venom by correlating coagulation bioassay peaks with HT venomics PSC data retrieved from database searches using the UniProt database and VenomZone from the Swiss-Prot group of the SIB Swiss Institute of Bioinformatics. Platelet aggregation inhibition toxin: PAIT, Hemostasis impairing toxin: HIT, Blood coagulation cascade activation: BCCA, Fibrinogenolytic activity: FA, Platelet aggregation activating toxin: PAAT, Blood coagulation cascade inhibition: BCCI, Hydrolase: HYD.

Bothrops asper					
Bioassay peak timepoint (Min)	Observed effect	Protein ID	Family	Subfamily, Sub-Subfamily	Theoretical effect(s)
20	Procoagulation	BOTAS_VM1B1	SVMP	P-I	FA, HIT, HEM
		BOTMO_VM11	SVMP	P-I	FA, HEM
		BOTPA_VM1B1	SVMP	P-I	FA, HIT
		BOTPA_VM1B2	SVMP	P-I	FA, HIT
		BOTPA_VM1N	SVMP	P-I	FA
		BOTLC_VM1LA	SVMP	P-I	PAIT, HIT
		BOTAS_VM3BA	SVMP	P-III, P-IIIa	BCCA
33	Procoagulation	BOTPA_VM1B1	SVMP	P-I	FA, HIT
		BOTJA_SLEB	Snaclec		PAAT, HIT
42	Procoagulation	BOTJA_VSP14	SVSP	Peptidase S1	FA, HIT
		BOTAT_OXLA	LAO	FIG1	PAAT, HIT
44	Procoagulation	BOTAT_VSPF	SVSP	Peptidase S1	BCCA, HIT
66	Anticoagulation	BOTAS_PA2B3	PLA2	Group 2, D49	PAIT, MYT
		BOTJR_PA2B2	PLA2	Group 2, D49	BCCI, HIT
		BOTAS_PA2A2	PLA2	Group 2, D49	HYD
		BOTJA_VM3B2	SVMP	P-III, P-IIIa	PAIT

Table S9. Overview of the tentatively identified bioactive coagulopathic toxins in *Bothrops atrox* venom by correlating coagulation bioassay peaks with HT venomics PSC data retrieved from database searches using the UniProt database and VenomZone from the Swiss-Prot group of the SIB Swiss Institute of Bioinformatics. Platelet aggregation inhibition toxin: PAIT, Hemostasis impairing toxin: HIT, Blood coagulation cascade activation: BCCA, Fibrinogenolytic activity: FA, Platelet aggregation activating toxin: PAAT, Hydrolase: HYD. *Monomeric form inhibits platelet aggregation and dimeric form results in platelet aggregation.

Bothrops atrox					
Bioassay peak timepoint (Min)	Observed effect	Protein ID	Family	Subfamily, Sub-Subfamily	Theoretical effect(s)
32	Procoagulation	BOTAT_OXLA	LAO	FIG1	PAAT, HIT
		BOTLC_OXLA	LAO	FIG1	PAAT
36	-	-	-		-
42	Procoagulation	BOTJA_VSP14	SVSP	Peptidase S1	FA
45	-	-	-	-	-
50	-	-	-	-	-
56	Procoagulation	BOTJA_VSPA	SVSP	Peptidase S1	PAAT, BCCA
		BOTAL_VSPTL	SVSP	Peptidase S1	HIT
		BOTAS_VM3BA	SVMP	P-III, P-IIIa	BCCA, FA
66	Anticoagulation	BOTJA_VM3JA	SVMP	P-III, P-IIIb	HEM, FA, PAAT, PAIT*
		BOTIN_VM36A	SVMP	P-III, P-IIIa	HIT
		BOTAT_VM11	SVMP	P-I	PAIT, HEM, FA
		BOTAS_PA2B3	PLA2	Group 2, D49	MYT
		BOTAS_PA2A2	PLA2	Group 2, D49	HYD

7. Transcriptomic bioactivity determination

Table S10. Overview of the tentatively identified bioactive coagulopathic toxins in *Bothrops asper* venom by correlating coagulation bioassay peaks with HT venomics PSC data retrieved from database searches using the additional transcriptomics databases.

***Bothrops asper* Transcriptomics**			
Timepoint	Transcriptomics code	Protein abbreviation/Family	Full protein name
20	A0A1L8D646	SVMP	Snake venom metalloproteinase
	ADO21504.1	ADO	2-aminoethanethiol dioxygenase
	ABB76281	SVMP	Snake venom metalloproteinase
	1ND1	SVMP	Snake venom metalloproteinase
33	A0A1L8D609	BATXCTL45	Batroxrhagin C-type Lectin
	A0A1L8D6D7	BATXCTL44	Batroxrhagin C-type Lectin
	A0A1L8D653	BATXCTL9	Batroxrhagin C-type Lectin
42	A0A1L8D5V4	BATXSVSP13	Batroxrhagin SVSP
	OXLA_BOTAT	OXLA	L-amino-oxidase
	A0A1L8D615	MAO	Amine oxidase
44	A0A977TFW8	SVSP	atroxobin
	VSPF_BOTAT	SVSP	Batroxobin
66	5TFV	PLA_2	Basic phospholipase A_2 myotoxin I
	P20474	PLA_2	Basic phospholipase A_2 myotoxin I
	A0A1L8D605	BATXPLA6	PLA_2 homolog of MtxIII
	ATP66581	PLA_2	Acidic myotoxic phospholipase A_2

Table S11. Overview of the tentatively identified bioactive coagulopathic toxins in *Bothrops atrox* venom by correlating coagulation bioassay peaks with HT venomics PSC data retrieved from database searches using the additional transcriptomics databases.

***Bothrops atrox* Transcriptomics**			
Timepoint	Transcriptomics code	Protein abbreviation/Family	Full protein name
32	A0A1L8D615	MAO	Amine oxidase
	OXLA_BOTAT	OXLA	L-amino-oxidase
36	P20474	PLA_2	Basic phospholipase A_2 myotoxin I
42	A0A1L8D5V4	BATXSVSP13	Batroxrhagin SVSP
	A0A1L8D654	BATXCTL20	Batroxrhagin C-type Lectin
45	-	-	-
50	BOTAT_VM32_3	SVMP	Batroxstatin-2
56	A0A1L8D668	BATXSVSP2	Batroxrhagin SVSP
	A0A0K2JNB8	SVMP	Snake venom metalloproteinase
	A0A1L8D5V7	SVMP	Snake venom metalloproteinase
66	A0A0K2JNB8	SVMP	Snake venom metalloproteinase
	A0A1S6K7T1	SVMP	Snake venom metalloproteinase
	A0A1L8D605	BATXPLA6	Batroxrhagin PLA_2
	A0A1L8D655	BATXSVMPI3	Batroxrhagin SVSP
	5TFV	PLA_2	Basic phospholipase A_2 myotoxin I
	A0A1L8D5Y9	SVMP	Snake venom metalloproteinase
	A0A1L8D5Y0	SVMP	Snake venom metalloproteinase

Bothrops atrox Transcriptomics			
Timepoint	Transcriptomics code	Protein abbreviation/Family	Full protein name
	A0A1L8D6B3	BATXCTL41	Batroxrhagin C-type Lectin

Table S12. Overview of the tentatively identified bioactive coagulopathic toxins in *Bothrops jararaca* venom by correlating coagulation bioassay peaks with HT venomics PSC data retrieved from database searches using the additional transcriptomics databases.

Bothrops jararaca Transcriptomics			
Timepoint	Transcriptomics code	Protein abbreviation/Family	Full protein name
23	-	-	-
30	21ed_SVMP_BJAR	SVMP	Snake venom metalloproteinase
	CTLX1_BJAR	CTL	C-type Lectin
36	02ed_SVMP_BJAR	SVMP	Snake venom metalloproteinase
	04edREV_SVMP_BJAR	SVMP	Snake venom metalloproteinase
	21ed_SVMP_BJAR	SVMP	Snake venom metalloproteinase
66	03ed_SVMP_BJAR	SVMP	Snake venom metalloproteinase
	CTL07_BJAR	CTL	C-type Lectin
	CTL24_BJAR	CTL	C-type Lectin

Table S13. Overview of the tentatively identified bioactive coagulopathic toxins in *Bothrops neuwiedi* venom by correlating coagulation bioassay peaks with HT venomics PSC data retrieved from database searches using the additional transcriptomics databases.

Bothrops neuwiedi Transcriptomics			
Timepoint	Transcriptomics code	Protein abbreviation/Family	Full protein name
24	ADO21504.1	SVMP	Snake venom metalloproteinase
	A0A1L8D600	BATXSVMPII1	Batroxrhagin SVMP
	ADO21505.1	SVMP	Snake venom metalloproteinase
	A0A1L8D5Z6	BATXSVMPII3	Batroxrhagin SVMP
	A0A1L8D5W7	SVMP	Snake venom metalloproteinase
28	ADO21504.1	SVMP	Snake venom metalloproteinase
	A0A1L8D600	BATXSVMPII1	Batroxrhagin SVMP
	A0A1L8D613	BATXCTL46	Batroxrhagin C-type Lectin
	A0A1L8D646	SVMP	Snake venom metalloproteinase
	A0A1L8D657	BATXCTL9	Batroxrhagin C-type Lectin
46	-	-	-
50	-	-	-
62	P0DUN6.1	PLA_2	Basic phospholipase A_2 homolog BnuTX-I
	A0A1L8D611	BATXPLA3	PLA_2 homolog of MtxIII
66	VM11_BOTAT	SVMP	Snake venom metalloproteinase atroxlysin-1
